# Computational design of SARS-CoV-2 peptide binders with better predicted binding affinities than human ACE2 receptor

**DOI:** 10.1038/s41598-021-94873-3

**Published:** 2021-08-02

**Authors:** Thassanai Sitthiyotha, Surasak Chunsrivirot

**Affiliations:** 1 Structural and Computational Biology Research Unit, Department of Biochemistry, Faculty of Science, Chulalongkorn University, Pathumwan, Bangkok, 10330 Thailand; 2 Department of Biochemistry, Faculty of Science, Chulalongkorn University, Pathumwan, Bangkok, 10330 Thailand

**Keywords:** Peptides, Protein design

## Abstract

SARS-CoV-2 is coronavirus causing COVID-19 pandemic. To enter human cells, receptor binding domain of S1 subunit of SARS-CoV-2 (SARS-CoV-2-RBD) binds to peptidase domain (PD) of angiotensin-converting enzyme 2 (ACE2) receptor. Employing peptides to inhibit binding between SARS-CoV-2-RBD and ACE2-PD is a therapeutic solution for COVID-19. Previous experimental study found that 23-mer peptide (SBP1) bound to SARS-CoV-2-RBD with lower affinity than ACE2. To increase SBP1 affinity, our previous study used residues 21–45 of α1 helix of ACE2-PD (SPB25) to design peptides with predicted affinity better than SBP1 and SPB25 by increasing interactions of residues that do not form favorable interactions with SARS-CoV-2-RBD. To design SPB25 with better affinity than ACE2, we employed computational protein design to increase interactions of residues reported to form favorable interactions with SARS-CoV-2-RBD and combine newly designed mutations with the best single mutations from our previous study. Molecular dynamics show that predicted binding affinities of three peptides (SPB25_Q22R_, SPB25_F8R/K11W/L25R_ and SPB25_F8R/K11F/Q22R/L25R_) are better than ACE2. Moreover, their predicted stabilities may be slightly higher than SBP1 as suggested by their helicities. This study developed an approach to design SARS-CoV-2 peptide binders with predicted binding affinities better than ACE2. These designed peptides are promising candidates as SARS-CoV-2 inhibitors.

## Introduction

The severe acute respiratory syndrome coronavirus 2 (SARS-CoV-2) is responsible for the coronavirus disease (COVID-19) pandemic that has caused large numbers of cases and deaths globally. SARS-CoV-2 consists of envelope (E), membrane (M), nucleocapsid (N), and spike (S) proteins^1,2^. The spike proteins of SARS-CoV-2 contain two subunits including S1 and S2 subunits that are responsible for fusion and entry of the virus into human cells. The receptor binding domain (RBD) of S1 subunit initially binds to the peptidase domain (PD) of angiotensin-converting enzyme 2 (ACE2) receptor of human cells, and the S2 subunit is responsible for the membrane fusion^3–8^. The α1-helix of the ACE2 peptidase domain (ACE2-PD) is a main recognition binding site of RBD of SARS-CoV-2 (SARS-CoV-2-RBD). The α2-helix and the linker of the β3- and β4-sheets also contribute to the binding of SARS-CoV-2-RBD^6,9^.

To control SARS-CoV-2 infections, various potential therapeutics have been explored such as neutralizing antibodies, small molecules and peptide inhibitors^10–19^. Disrupting the protein–protein binding interfaces of SARS-CoV-2-RBD and ACE2-PD to prevent coronavirus entry in human cells is a promising therapeutic solution for COVID-19. As alternatives to small molecules, peptides can potentially be used as inhibitors to disrupt the binding between SARS-CoV-2-RBD and ACE2-PD because peptides have a large number of functional groups for favorable interactions at the binding interface and structural compatibility with the target protein that leads to less potential to interfere with normal biological processes^20,21^. An example of a peptide inhibitor that is currently used as medicine is Enfuvirtide that has been clinically approved as a peptide inhibitor to inhibit HIV entry^22^.

Furthermore, since SARS-CoV-2 infection usually starts in the nasal cavity, where coronavirus replicates in this area for days and later spreads to lower respiratory tract^23^, delivery of a high dose of a viral inhibitor into the nose and the respiratory system could provide protection and treatment for early infection that can be very beneficial especially for frontline healthcare workers and essential workers^15^. Although monoclonal antibodies are in development for COVID-19 treatment^17–19,24^, they may not be effectively administered via intranasal delivery because of their large sizes, low binding site density^15^, and potential issue with antibody-dependent disease enhancement^25–27^. Peptides or small proteins with high binding affinity to SARS-CoV-2-RBD could have advantages over antibodies for direct delivery into the respiratory system via intranasal administration, nebulization or dry powder aerosol because of their smaller sizes and higher density of inhibitory domains^15^. Previous study also reported that small proteins with high binding affinity to the influenza hemagglutinin when delivered intranasally can provide prophylactic and therapeutic protection in rodent models of lethal influenza infection^28^.

Computational techniques have been used to design peptides that could potentially bind to SARS-CoV-2-RBD^29–34^. The previous experimental study found that the 23-mer peptide binder (SBP1) that was derived from the α1 helix (residues 21–43) of ACE2-PD bound to SARS-CoV-2-RBD (*K*_D_ = 47 nM)^32^ has lower binding affinity than ACE2 (*K*_D_ = 14.7 nM)^35^ and it could potentially be used as a peptide inhibitor of SARS-CoV-2. To increase the binding affinity of SBP1, our previous study^36^ employed computational protein design and molecular dynamics (MD) to design 25-mer peptide binder (SPB25) of SARS-CoV-2 based on residues 21–45 of the α1 helix of ACE2-PD. The design strategy of our previous study was to increase favorable interactions and avoid disrupting existing favorable interactions by designing only residues that have not been reported to form favorable interactions with SARS-CoV-2-RBD and allowing them to be any of standard amino acids except G and P. The results show that five designed peptides (SPB25_F8N_, SPB25_F8R_, SPB25_L25R_, SPB25_F8N/L25R_, and SPB25_F8R/L25R_) have better predicted binding affinities to SARS-CoV-2-RBD than SPB25 and SBP1. However, the binding affinity to SARS-CoV-2-RBD of SPB25 can be further enhanced to improve its effectiveness as a therapeutic solution for COVID19.

The aim of this work is to use computational protein design (Rosetta) and MD (AMBER) to design 25-mer peptide binders with better predicted binding affinities to SARS-CoV-2-RBD than human ACE2 receptor. Our design strategy is to increase the binding affinity of residues that were previously reported to form favorable interactions between residues 21–45 of ACE2 and SARS-CoV-2-RBD^29,37^ and combine the newly designed single mutations with the best designed single mutations from our previous study to further enhance the binding affinities of the designed peptides. The designed peptides with better predicted binding affinities to SARS-CoV-2-RBD than human ACE2 receptor are promising candidates as potential SARS-CoV-2 inhibitors.

## RESULTS

### Computational design of SARS-CoV-2-RBD peptide binders

**Figure 1:**
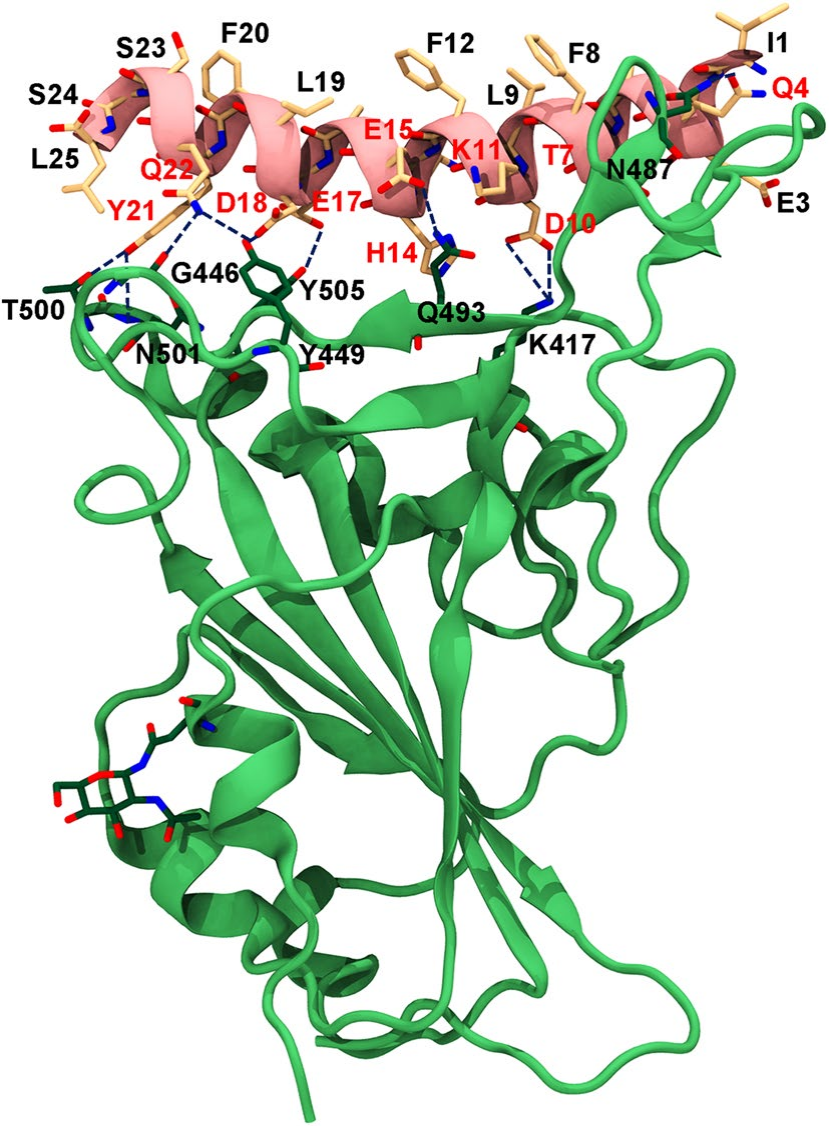
The structure of SPB25/SARS-CoV-2-RBD complex that was used as a design template. SPB25 and SARS-CoV-2-RBD are colored in pink and green, respectively. The designed positions (Q4, T7, D10, K11, H14, E15, E17, D18, Y21 and Q22) are labelled in red.

The structure of the design template of SPB25 bound to SARS-CoV-2-RBD (Fig. 1) was obtained from the crystal structure of the α1 helix of ACE2 peptidase domain (ACE2-PD) bound to SARS-COV-2-RBD (PDB ID: 6M0J)^37^. In this study, the design strategy is to increase the binding affinity of residues that were previously reported to form favorable interactions between residue 21–45 of ACE2 and SARS-CoV-2-RBD^29,37^ and then combine the newly designed single mutations with the best designed single mutations from our previous study to further enhance the binding affinities of the designed peptides so that their predicted binding affinities are better than ACE2; our previous work designed the residues that have not been reported to form favorable interactions with SARS-CoV-2-RBD to increase favorable interactions of these residues and avoid disrupting existing favorable interactions. In this study, Rosetta was employed to design SARS-CoV-2-RBD peptide binders, and the designed positions were selected from the residues that were previously reported to form favorable interactions with SARS-CoV-2-RBD^29,37^ and their side chains could potentially form favorable interactions upon mutations with SARS-CoV-2-RBD. In this study, Q4 (24), T7 (27), D10 (30), K11 (31), H14 (34), E15 (35), E17 (37), D18 (38), Y21 (41) and Q22 (42) were selected based on these criteria. Each designed position was allowed to be any of standard amino acids except G and P because G and P occur infrequently in an α-helix. P also can cause a destabilizing kink in a helix structure^38^. The total of 156 designed peptides with single mutation were obtained from Rosetta (Table S1). Ten designed peptides with better ΔG_bind (Rosetta)_ than SPB25 (ΔΔG_bind (Rosetta)_ < 0 REU) were selected for MD simulations to validate whether their predicted binding affinities by the more accurate Molecular Mechanics-Generalized Born Surface Area (MM-GBSA) method^39–41^ (ΔG_bind (MM-GBSA)_) were better than that of SPB25 (ΔΔG_bind (MM-GBSA)_ < 0 kcal/mol). These designed peptides are SPB25_T7I,_ SPB25_T7V,_ SPB25_K11F,_ SPB25_K11W_, SPB25_H14V,_ SPB25_E15L,_ SPB25_E17F,_ SPB25_E17W_, SPB25_D18E_ and SPB25_Q22R_.

### Validation by MD

MD simulations were performed on ten complex structures of ten designed peptides with single mutations binding to SARS-CoV-2-RBD, and the MM-GBSA method was employed to calculate their ΔG_bind (MM-GBSA)_ values to determine whether their predicted binding affinities were better than SPB25. Their predicted binding affinities were compared to the predicted binding affinities of ACE2 (− 71.2 ± 0.4 kcal/mol), SBP1 (− 55.1 ± 0.4 kcal/mol) and SPB25 (− 60.3 ± 0.4 kcal/mol) from our previous study^36^; the experimental *K*_D_ values of SBP1 and ACE2 are 47 and 14.7 nM, respectively^32,35^. The Root Mean Square Deviation (RMSD) values of all atoms and backbone atoms were calculated to monitor the stabilities of all systems (Figure S1). All systems were likely to reach equilibrium around 80 ns. Therefore, the 80–100 ns trajectories of all systems were selected for further analyses.

The MM-GBSA method was employed to calculate ΔG_bind (MM-GBSA)_ to predict the binding affinities of all systems during the 80–100 ns trajectories (Table 1). Out of ten designed peptides with single mutation, SPB25_K11F,_ SPB25_K11W_ and SPB25_Q22R_ have better ΔG_bind (MM-GBSA)_ than SPB25 with ΔΔG_bind (MM-GBSA)_ of − 11.3 ± 0.7, − 2.9 ± 0.6 and − 15.0 ± 0.6 kcal/mol, respectively. These three designed single mutations were combined with the best three single mutations from our previous study (SPB25_F8N_, SPB25_F8R_ and SPB25_L25R_)^36^ to create designed peptides with double, triple and quadruple mutations. The total of 11, 12 and 4 designed peptides with double, triple and quadruple mutations were additionally constructed using Rosetta and subjected to MD validation. In this study, the designed peptides with double mutation did not include SPB25_F8N/L25R_ and SPB25_F8R/L25R_ because they were already simulated, and their values of ΔG_bind (Rosetta)_ were already reported in our previous work. As shown in Table 1, the values of ΔG_bind (Rosetta)_ of all 27 designed peptides with double, triple and quadruple mutations are better than that of SPB25 (ΔΔG_bind (Rosetta)_ < 0 REU). In terms of the binding affinities of designed peptides with double mutations, the values of ΔΔG_bind (MM-GBSA)_ of SPB25_F8N/K11W_, SPB25_F8R/K11F_, SPB25_F8R/K11W_, SPB25_F8R/Q22R_, SPB25_K11F/L25R_ and SPB25_K11W/L25R_ are better than that of SPB25 with the ΔΔG_bind (MM-GBSA)_ values of − 6.5 ± 0.6, − 9.4 ± 0.6, − 9.2 ± 0.6, − 3.6 ± 0.5, − 2.8 ± 0.6 and − 4.0 ± 0.6 kcal/mol, respectively. For designed peptides with triple mutations, SPB25_F8N/K11F/L25R,_ SPB25_F8N/K11W/L25R,_ SPB25_F8R/K11W/L25R_ and SPB25_K11W/Q22R/L25R_ have better ΔG_bind (MM-GBSA)_ than SPB25 with ΔΔG_bind (MM-GBSA)_ of − 0.3 ± 0.6, − 1.4 ± 0.6, − 14.7 ± 0.5 and − 7.5 ± 0.6 kcal/mol, respectively. In terms of the designed peptides with quadruple mutations, the ΔG_bind (MM-GBSA)_ values of SPB25_F8R/K11F/Q22R/L25R_ and SPB25_F8R/K11W/Q22R/L25R_ are better than those of SPB25 with ΔΔG_bind (MM-GBSA)_ of − 11.9 ± 0.6 and − 7.1 ± 0.6 kcal/mol, respectively. Moreover, the predicted binding affinities of these designed peptides are better than that of SBP1, which is the experimentally proven peptide binder of SARS-CoV-2-RBD^32^. Most importantly, the predicted binding affinities of SPB25_Q22R_ (ΔG_bind (MM-GBSA)_ =  − 75.3 ± 0.5 kcal/mol), SPB25_F8R/K11W/L25R_ (ΔG_bind (MM-GBSA)_ =  − 75.0 ± 0.3 kcal/mol) and SPB25_F8R/K11F/Q22R/L25R_ (ΔG_bind (MM-GBSA)_ =  − 72.2 ± 0.4 kcal/mol) are better than that of ACE2 (ΔG_bind (MM-GBSA)_ =  − 71.2 ± 0.4 kcal/mol), while that of SPB25_K11F_ (ΔG_bind (MM-GBSA)_ of − 71.6 ± 0.6 kcal/mol) is about the same as that of ACE2.

**Table 1:** The predicted binding free energies to SARS-CoV-2-RBD of ACE2, SBP1, SPB25 and designed peptides that were selected for MD simulations, as calculated by Rosetta and the MM-GBSA method.

System	ΔΔG_bind (Rosetta)_^a^ (REU)	ΔG_bind (MM-GBSA)_ (kcal/mol)	ΔΔG_bind (MM-GBSA)_^b^ (kcal/mol)
ACE2^36^	–	− 71.2 ± 0.4	− 10.9 ± 0.6
SBP1^36^	–	− 55.1 ± 0.4	5.2 ± 0.6
SPB25^36^	0.0	− 60.3 ± 0.4	0.0 ± 0.6
SPB25_T7I_	− 0.3	− 59.2 ± 0.3	1.1 ± 0.5
SPB25_T7V_	− 0.4	− 50.9 ± 0.3	9.4 ± 0.5
SPB25_K11F_	− 0.4	− 71.6 ± 0.6	− 11.3 ± 0.7
SPB25_K11W_	− 2.2	− 63.2 ± 0.4	− 2.9 ± 0.6
SPB25_H14V_	− 0.1	− 58.2 ± 0.5	2.1 ± 0.6
SPB25_E15L_	− 0.9	− 51.7 ± 0.4	8.6 ± 0.6
SPB25_E17F_	− 0.9	− 47.7 ± 0.4	12.6 ± 0.6
SPB25_E17W_	− 3.1	− 57.8 ± 0.5	2.5 ± 0.6
SPB25_D18E_	− 0.5	− 55.4 ± 0.4	4.9 ± 0.6
SPB25_Q22R_	− 0.4	− 75.3 ± 0.5	− 15.0 ± 0.6
SPB25_F8N/K11F_	− 3.4	− 56.8 ± 0.4	3.5 ± 0.6
SPB25_F8N/K11W_	− 6.6	− 66.8 ± 0.5	− 6.5 ± 0.6
SPB25_F8N/Q22R_	− 3.0	− 58.0 ± 0.3	2.3 ± 0.5
SPB25_F8R/K11F_	− 4.9	− 69.7 ± 0.5	− 9.4 ± 0.6
SPB25_F8R/K11W_	− 5.6	− 69.5 ± 0.4	− 9.2 ± 0.6
SPB25_F8R/Q22R_	− 1.5	− 63.9 ± 0.3	− 3.6 ± 0.5
SPB25_K11F/Q22R_	− 3.9	− 53.7 ± 0.6	6.6 ± 0.7
SPB25_K11F/L25R_	− 3.6	− 63.1 ± 0.5	− 2.8 ± 0.6
SPB25_K11W/Q22R_	− 4.0	− 44.0 ± 0.4	16.3 ± 0.6
SPB25_K11W/L25R_	− 3.6	− 64.3 ± 0.4	− 4.0 ± 0.6
SPB25_Q22R/L25R_	− 1.8	− 47.7 ± 0.4	12.6 ± 0.6
SPB25_F8N/K11F/Q22R_	− 6.3	− 48.5 ± 0.6	11.8 ± 0.7
SPB25_F8N/K11F/L25R_	− 3.9	− 60.6 ± 0.5	− 0.3 ± 0.6
SPB25_F8N/K11W/Q22R_	− 5.8	− 56.5 ± 0.5	3.8 ± 0.6
SPB25_F8N/K11W/L25R_	− 3.8	− 61.7 ± 0.4	− 1.4 ± 0.6
SPB25_F8N/Q22R/L25R_	− 2.0	− 58.1 ± 0.4	2.2 ± 0.6
SPB25_F8R/K11F/Q22R_	− 4.5	− 59.3 ± 0.4	1.0 ± 0.6
SPB25_F8R/K11F/L25R_	− 2.4	− 58.1 ± 0.4	2.2 ± 0.6
SPB25_F8R/K11W/Q22R_	− 3.6	− 52.9 ± 0.4	7.4 ± 0.6
SPB25_F8R/K11W/L25R_	− 5.1	− 75.0 ± 0.3	− 14.7 ± 0.5
SPB25_F8R/Q22R/L25R_	− 0.2	− 60.2 ± 0.4	0.1 ± 0.6
SPB25_K11F/Q22R/L25R_	− 2.5	− 53.7 ± 0.4	6.6 ± 0.6
SPB25_K11W/Q22R/L25R_	− 1.2	− 67.8 ± 0.5	− 7.5 ± 0.6
SPB25_F8N/K11F/Q22R/L25R_	− 6.8	− 58.7 ± 0.6	1.6 ± 0.7
SPB25_F8N/K11W/Q22R/L25R_	− 4.0	− 60.2 ± 0.4	0.1 ± 0.6
SPB25_F8R/K11F/Q22R/L25R_	− 2.8	− 72.2 ± 0.4	− 11.9 ± 0.6
SPB25_F8R/K11W/Q22R/L25R_	− 3.9	− 67.4 ± 0.4	− 7.1 ± 0.6

^a^The difference between ΔG_bind (Rosetta)_ of a system and that of SPB25.

^b^The difference between ΔG_bind (MM-GBSA)_ of a system and that of SPB25.

### The binding free energy components of designed peptides with predicted binding affinity to SARS-CoV-2-RBD better than or similar to ACE2

**Figure 2:**
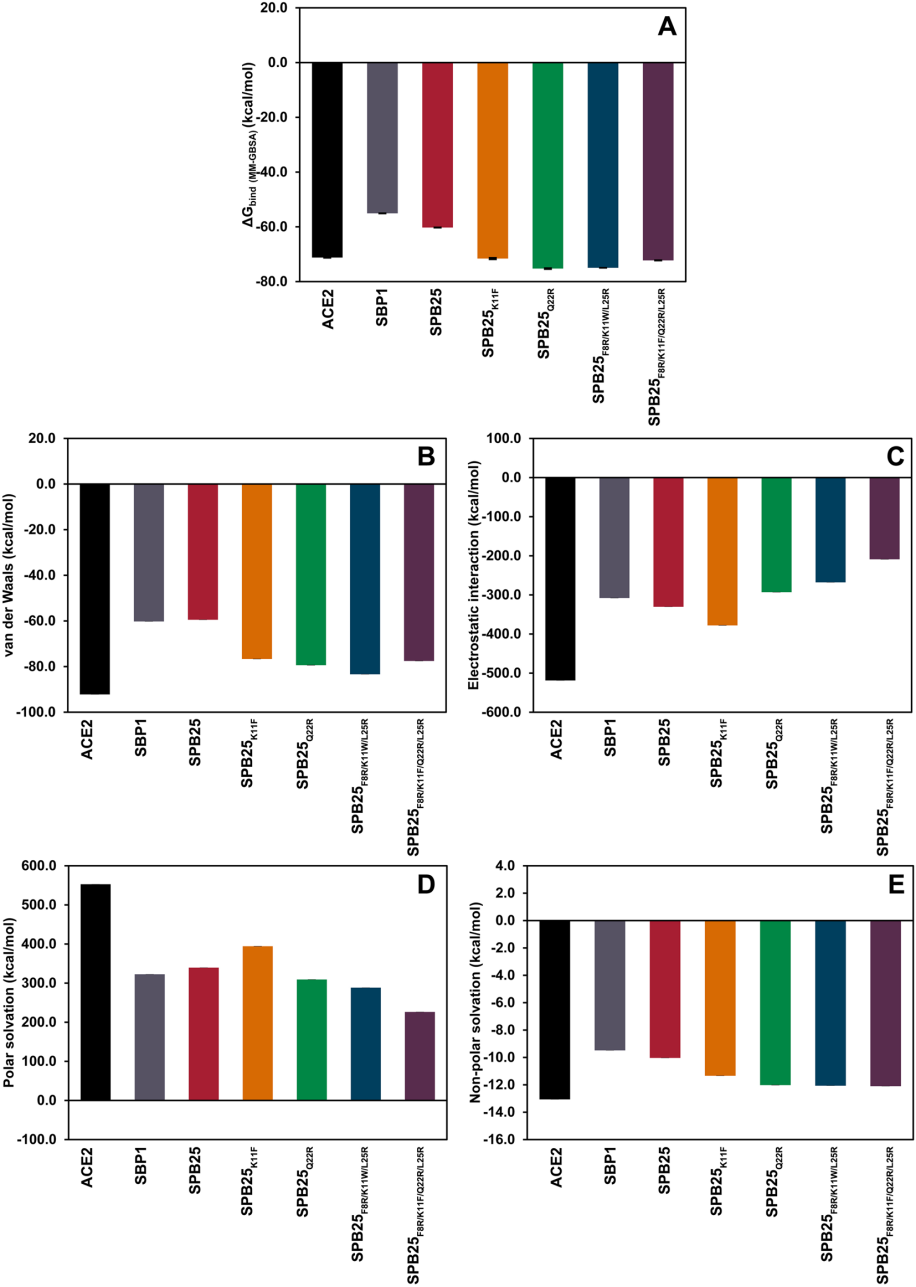
The binding free energy components of ACE2/SARS-CoV-2-RBD^36^, SBP1/SARS-CoV-2-RBD^36^, SPB25/SARS-CoV-2-RBD^36^ and designed peptides/SARS-CoV-2-RBD. (**A**) ΔG_bind (MM-GBSA)_, (**B**) van der Waals energy, (**C**) electrostatic interaction, (**D**) polar solvation and (**E**) non-polar solvation.

Figure 2 shows binding energy components of the four designed peptides with predicted binding affinities better than or similar to ACE2 (Fig. 2) as compared to those of ACE2, SBP1 and SPB25. The electrostatic interaction terms are the main components contributing to the favorable predicted binding affinities of SPB25_K11F,_ SPB25_Q22R_, SPB25_F8R/K11W/L25R_ and SPB25_F8R/K11F/Q22R/L25R_ to SARS-CoV-2-RBD. The van der Waals energy and non-polar solvation terms also contributes favorably to the predicted binding affinity. However, the polar solvation terms have unfavorable contribution to the predicted binding affinity.

As shown in Fig. 2 and Table 1, SPB25_Q22R_ is the designed peptide with the best predicted binding affinity with the ΔG_bind (MM-GBSA)_ value of − 75.3 ± 0.5 kcal/mol. Its predicted binding affinity is better than those of ACE2, SBP1 and SPB25 by − 4.1 ± 0.6, − 20.2 ± 0.6 and − 15.0 ± 0.6 kcal/mol, respectively. The favorable binding of SPB25_Q22R_ to SARS-CoV-2-RBD is mostly caused by the increase in the favorable van der Waals energy and non-polar solvation terms as well as the decrease in unfavorable polar solvation term as compared to those of SBP1 and SPB25. The favorable electrostatic interaction term of SPB25_Q22R_ is worse than those of SBP1 and SPB25. The predicted binding affinity of SPB25_K11F_ is better than those of SBP1 and SPB25 and similar to that of ACE2. The favorable binding of SPB25_K11F_ to SARS-CoV-2-RBD is mostly caused by the increase in the favorable van der Waals energy, electrostatic interaction terms and non-polar solvation terms as compared to those of SBP1 and SPB25. The unfavorable polar solvation term of SPB25_K11F_ is worse than that of SBP1 and SPB25. The predicted binding affinities of SPB25_F8R/K11W/L25R_ and SPB25_F8R/K11F/Q22R/L25R_ are better than those of SBP1, SPB25 and ACE2. The favorable binding of SPB25_F8R/K11W/L25R_ and SPB25_F8R/K11F/Q22R/L25R_ to SARS-CoV-2-RBD is mostly caused by the increase in the favorable van der Waals energy and non-polar solvation terms as well as the decrease in unfavorable polar solvation terms as compared to those of SBP1 and SPB25. However, the favorable electrostatic interaction terms of these two designed peptides are worse than those of SBP1 and SPB25. The predicted binding affinities to SARS-CoV-2-RBD of the three designed peptides are better than that of ACE2 because their unfavorable polar solvation terms are substantially lower than that of ACE2 although their favorable van der Waals, electrostatic interaction and non-polar solvation terms are worse than that of ACE2.

### Identification of important binding residues of designed peptides with predicted binding affinity to SARS-CoV-2-RBD better than or similar to ACE2

**Figure 3:**
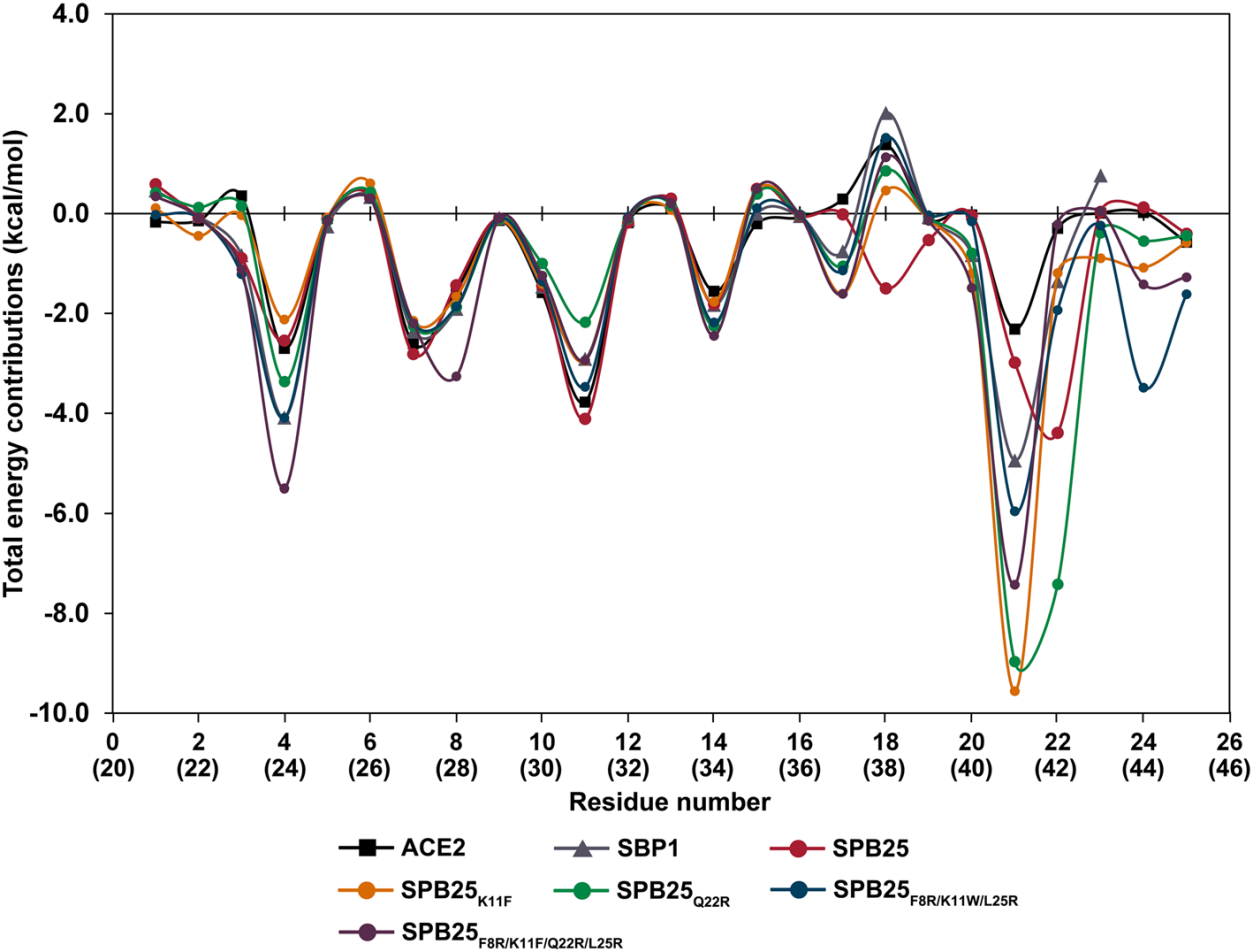
Per-residue free energy decomposition of ACE2^36^, SBP1^36^, SPB25^36^ and designed peptides in binding to SARS-CoV-2-RBD. The residue number of ACE2 is in parenthesis.

To identify important binding residues to SARS-CoV-2-RBD of four designed peptides with predicted binding affinities better than or similar to ACE2, per-residue free energy decomposition was calculated and shown in Fig. 3. An important binding residue was defined to be a residue with the total energy contribution better than − 1.0 kcal/mol^42^. Overall, the number of important binding residues of SPB25_K11F_ (11), SPB25_Q22R_ (8), SPB25_F8R/K11W/L25R_ (12) and SPB25_F8R/K11F/Q22R/L25R_ (12) were predicted to be relatively similar or more than those of SBP1 (8), SPB25 (9) and residues 21–45 of the α1 helix of ACE2 (7)^36^. Overall, four residues of all designed peptides were predicted to have high binding affinity (better than − 2.0 kcal/mol) such as Y21 (the best binding residue), Q4, T7, and K11/F11/W11. Additionally, H14 of SPB25_Q22R_, SPB25_F8R/K11W/L25R_ and SPB25_F8R/K11F/Q22R/L25R_, R22 of SPB25_Q22R_ as well as S24 of SPB25_F8R/K11W/L25R_ were also predicted to have high binding affinity to SARS-CoV-2-RBD.

In terms of per-residue free energy decomposition of SPB25_K11F_, the K11F mutation was predicted to unfavorably decrease the total energy contribution of this residue from − 3.8 and − 4.1 kcal/mol in ACE2 and SPB25, respectively, to − 2.9 kcal/mol in SPB25_K11F_. However, this mutation caused significant favorable changes to the total energy contributions of other residues. The total energy contributions of other residues such as F8, E17, F20, Y21 and S24 were substantially increased from − 1.5, 0.3, 0.0, − 2.3 and 0.0 kcal/mol in ACE2 and − 1.4, 0.0, 0.0, − 3.0 and 0.1 kcal/mol in SPB25 to − 1.7, − 1.6, − 1.2, − 9.6 and − 1.1 kcal/mol in SPB25_K11F_, respectively. Moreover, the total energy contribution of residues Q22 was favorably increased from − 0.3 kcal/mol in ACE2 to − 1.2 kcal/mol in SPB25_K11F_. For per-residue free energy decomposition of SPB25_Q22R_, the Q22R mutation was predicted to favorably increase the total energy contribution from − 0.3 and − 4.4 kcal/mol in ACE2 and SPB25, respectively, to − 7.4 kcal/mol in SPB25_Q22R_. Additionally, the total energy contributions of Q4, F8, H14, E17 and Y21 were favorably increased from − 2.7, − 1.5, − 1.6, 0.3 and − 2.3 kcal/mol in ACE2 and − 2.5, − 1.4, − 1.8, 0.0 and − 3.0 kcal/mol in SPB25 to − 3.4, − 1.9, − 2.3, − 1.1 and − 9.0 kcal/mol in SPB25_Q22R_, respectively.

In terms of the designed peptides with triple mutation, the F8R/K11W/L25R mutation was predicted to favorably increase the total energy contributions of residues 8 and 25 from − 1.5 and − 0.6 kcal/mol in ACE2 and − 1.4 and − 0.4 kcal/mol in SPB25 to − 1.9 and − 1.6 kcal/mol in SPB25_F8R/K11W/L25R,_ respectively. However, the total energy contribution of residue 11 was unfavorably decreased from − 3.8 and − 4.1 kcal/mol in ACE2 and SPB25, respectively, to − 3.5 kcal/mol in SPB25_F8R/K11W/L25R._ However, the total energy contributions of other residues such as E3, Q4, H14, E17, Y21 and S24 were favorably increased from 0.4, − 2.7, − 1.6, 0.3, − 2.3 and 0.0 kcal/mol in ACE2 and − 0.9, − 2.5, − 1.8, 0.0, − 3.0 and 0.1 kcal/mol in SPB25 to − 1.2, − 4.1, − 2.2, − 1.1, − 6.0 and − 3.5 kcal/mol in SPB25_F8R/K11W/L25R_, respectively. Additionally, the total energy contribution of residues Q22 was favorably increased from − 0.3 kcal/mol in ACE2 to − 1.9 kcal/mol in SPB25_F8R/K11W/L25R_.

In terms of the designed peptides with quadruple mutation, the F8R/K11F/Q22R/L25R mutation was predicted to favorably increase the total energy contributions of residues 8 and 25 from − 1.5 and − 0.6 kcal/mol in ACE2 and − 1.4 and − 0.4 kcal/mol in SPB25 to − 3.3 and − 1.3 kcal/mol in SPB25_F8R/K11F/Q22R/L25R_, respectively, while this quadruple mutation was predicted to unfavorably decrease the total energy contributions of residues 11 and 22 from − 3.8 and − 0.3 kcal/mol in ACE2 and − 4.1 and − 4.4 kcal/mol in SPB25 to − 2.9 and − 0.2 kcal/mol in SPB25_F8R/K11F/Q22R/L25R_, respectively. In addition, the total energy contributions of other residues such as E3, Q4, H14, E17, F20, Y21 and Y24 were favorably increased from 0.4, − 2.7, − 1.6, 0.3, 0.0, − 2.3 and 0.0 kcal/mol in ACE2 and − 0.9, − 2.5, − 1.8, 0.0, 0.0 − 3.0 and 0.1 kcal/mol in SPB25 to − 1.1, − 5.5, − 2.4, − 1.6, − 1.5, − 7.4 and − 1.4 kcal/mol in SPB25_F8R/K11F/Q22R/L25R_, respectively.

### Hydrogen bond and pi interactions of designed peptides with predicted binding affinities to SARS-CoV-2-RBD better than or similar to ACE2

**Figure 4:**
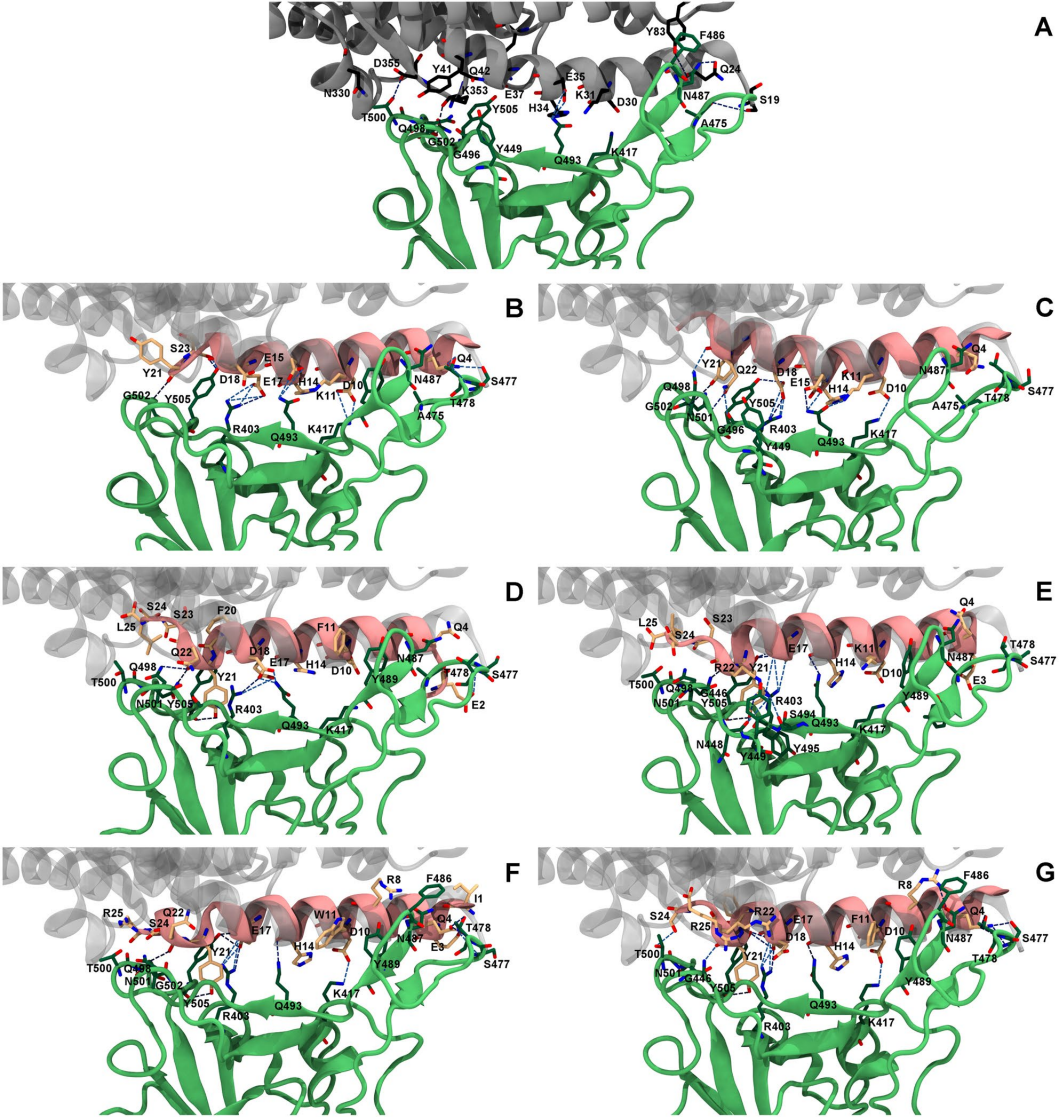
Key binding interactions between SARS-CoV-2-RBD (green) and (**A**) ACE2^36^, (**B**) SBP1^36^, (**C**) SPB25^36^, (**D**) SPB25_K11F_, (**E**) SPB25_Q22R_, (**F**) SPB25_F8R/K11W//L25R_ or (**G**) SPB25_F8R/K11F/Q22R/L25R_. The structures of SBP1, SPB25 and designed peptides (pink) were superimposed with ACE2 (grey). Key hydrogen bonds and salt bridges (hydrogen bond occupations > 25%) are shown in blue dashed lines. These structures are the structures closest to the average structures from the 80–100 ns MD trajectories.

To identify important hydrogen bonds and pi interactions for the binding to SARS-CoV-2-RBD of four designed peptides with predicted binding affinities better than or similar to ACE2, hydrogen bond occupations, pi–pi, cation–pi and sigma–pi interactions were analyzed as shown in Table 2 and Table S2. Key binding interactions are shown in Fig. 4. Overall, the binding positions and orientations of all designed peptides to SARS-CoV2-RBD are relatively similar to those of ACE2. In terms of the designed peptides with single mutation, the total numbers of predicted hydrogen bonds and pi interactions of SPB25_K11F_ are more than those of ACE2, SPB25 and SBP1, supporting the binding energy result that it has better predicted binding affinity to SARS-CoV-2-RBD than ACE2, SPB25 and SBP1. Residues E2, Q4, D10, H14, E17, D18, Y21, Q22, S23, S24 and L25 of SPB25_K11F_ were predicted to form hydrogen bonds with SARS-CoV-2-RBD. The mutated residue F11 of SPB25_K11F_ was predicted to form pi–pi interaction with Y489 of SARS-CoV-2-RBD, while F11 of ACE2, SPB25 and SBP1 were not predicted to form pi–pi interaction with SARS-CoV-2-RBD. Additionally, our study predicted one pi–pi (F20-Y505), two cation–pi interactions (Y21-R403:NH1 and Y21-R403:NH2) and one sigma–pi (Y21-Y505:HD2) interactions between SPB25_K11F_ and SARS-CoV-2-RBD. The total number of predicted hydrogen bonds of SPB25_Q22R_ is more than those of ACE2, SBP1 and relatively similar to that of SPB25, but the number of strong hydrogen bonds of SPB25_Q22R_ is more than that of SPB25. The total number of pi interactions of SPB25_Q22R_ is more than those of ACE2, SPB25 and SBP1. The mutated residue R22 of SPB25_Q22R_ was predicted to form one medium hydrogen bonds with the backbone of N448, three weak hydrogen bonds with G446 (backbone), N448 (backbone) and S494, and one very weak hydrogen bond with the backbone of Y495 of SARS-CoV-2-RBD. This mutated residue was also predicted to form two cation–pi interactions with SARS-CoV-2-RBD (R22:NH1-Y449 and R22:NH2-Y449). Other residues such as E3, Q4, D10, H14, E15, E17, Y21, S23, S24 and L25 of SPB25_Q22R_ were also predicted to form hydrogen bonds with SARS-CoV-2-RBD. Furthermore, there are four predicted cation–pi interactions (K11:NZ-Y489, H14-K417:NZ, Y21-R403:NH1 and Y21-R403:NH2,) and one predicted sigma–pi interaction (Y21-Y505:HD1) formed between SPB25_Q22R_ and SARS-CoV-2-RBD.

**Table 2:** Numbers of hydrogen bond and pi interactions of ACE2, SBP1, SPB25 and designed peptides contributing to SARS-CoV-2-RBD binding.

System	Number of hydrogen bonds				Residue that forms a hydrogen bond with SARS-CoV-2-RBD using its backbone or side chain	Interaction		
	Strong	Medium	Weak	Very weak		Pi–Pi	Cation–Pi	Sigma–Pi
ACE2^36^	2	3	2	25	S19, Q24, D30, K31, H34, E35, E37, Y41, Q42, Y83, N330, K353, D355	Y83-F486	H34-K417:NZ R393:NH1-Y505	K353:HA-Y505
SBP1^36^	1	2	11	14	Q4, D10, K11, H14, E15, E17, D18, Y21, S23	–	K11:NZ-Y489 H14-K417:NZ	–
SPB25^3,6^	1	4	11	20	Q4, D10, K11, H14, E15, D18, Y21, Q22	Y21-Y505	H14-K417:NZ	–
SPB25_K11F_	2	3	6	33	E2, Q4, D10, H14, E17, D18, Y21, Q22, S23, S24, L25	F11-Y489 F20-Y505	Y21-R403:NH1 Y21-R403:NH2	Y21-Y505:HD2
SPB25_Q22R_	2	4	8	22	E3, Q4, D10, H14, E15, E17, Y21, R22, S23, S24, L25	–	K11:NZ-Y489 H14-K417:NZ Y21-R403:NH1 Y21-R403:NH2 R22:NH1-Y449 R22:NH2-Y449	Y21-Y505:HD1
SPB25_F8R/K11W/L25R_	4	4	5	20	I1, E3, Q4, R8, D10, H14, E17, Y21, Q22, S24, R25	W11_(pyrrole)_-Y489 W11(_benzene_)-Y489	R8:NH2-F486 Y21-R403:NH1 Y21-R403:NH2	Y21-Y505:HD2
SPB25_F8R/K11F/Q22R/L25R_	4	6	6	16	Q4, R8, D10, H14, E17, D18, F20, Y21, S24, R25	F11-Y489 F20-Y505	R8:NH1-F486 R8:NH2-F486 H14-K417:NZ Y21-R403:NH1 Y21-R403:NH2	Y21-Y505:HD2

In terms of the designed peptides with triple mutation, the total number of predicted hydrogen bonds of SPB25_F8R/K11W//L25R_ is higher than those of ACE2 and SBP1 and lower than that of SPB25, but the number of strong hydrogen bonds of SPB25_F8R/K11W//L25R_ is more than that of SPB25. The predicted number of pi interactions of SPB25_F8R/K11W//L25R_ is higher than those of ACE2, SBP1 and SPB25. Furthermore, the mutated residue R8 of SPB25_F8R/K11W//L25R_ was predicted to form four very weak hydrogen bonds with N487 and Y489 of SARS-CoV-2-RBD, and the mutated residue R25 was predicted to form one very weak hydrogen bonds with T500 (backbone) of SARS-CoV-2-RBD, while F8 and L25 of ACE2, SPB25 and SBP1 were not predicted to form any hydrogen bonds with SARS-CoV-2-RBD. Moreover, I1, E3, Q4, D10, H14, E17, Y21, Q22 and S24 of SPB25_F8R/K11W//L25R_ were predicted to form hydrogen bonds with SARS-CoV-2-RBD. Additionally, the mutated residue R8 of SPB25_F8R/K11W//L25R_ was predicted to form cation–pi interaction with F486 of SARS-CoV-2-RBD, and the mutated residue W11 of SPB25_F8R/K11W//L25R_ was also predicted to form pi–pi interaction with Y489 of SARS-CoV-2-RBD, while F8 and K11 of ACE2 and SPB25 were not predicted to form pi interactions with SARS-CoV-2-RBD. Other residues were also predicted to form two cation–pi interactions (Y21-R403:NH1 and Y21-R403:NH2) and one sigma–pi interaction (Y21-Y505:HD2) between SPB25_F8R/K11W//L25R_ and SARS-CoV-2-RBD.

In terms of the designed peptides with quadruple mutations, the total number of predicted hydrogen bonds of SPB25_F8R/K11F/Q22R/L25R_ is higher than that of SBP1, lower than that of SPB25, and similar to that of ACE2, but the number of strong hydrogen bonds of SPB25_F8R/K11F/Q22R/L25R_ is higher than those of SBP1, SPB25 and ACE2. Moreover, the predicted number of pi interactions of SPB25_F8R/K11F/Q22R/L25R_ is higher than those of ACE2, SBP1 and SPB25. The mutated residue R8 of SPB25_F8R/K11F/Q22R/L25R_ was predicted to form one medium and two very weak hydrogen bonds with N487 of SARS-CoV-2-RBD, and the mutated residue R25 of SPB25_F8R/K11F/Q22R/L25R_ was predicted to form two very weak hydrogen bonds with the backbone of G446 of SARS-CoV-2-RBD, while F8 and L25 of ACE2, SPB25 and SBP1 were not predicted to form any hydrogen bonds with SARS-CoV-2-RBD. Other residues such as Q4, D10, H14, E17, D18, F20, Y21 and S24 of SPB25_F8R/K11F/Q22R/L25R_ were also predicted to form hydrogen bonds with SARS-CoV-2-RBD. Additionally, the mutated residue R8 of SPB25_F8R/K11F/Q22R/L25R_ was predicted to form two cation–pi interactions with F486 of SARS-CoV-2-RBD, and the mutated residue F11 of SPB25_F8R/K11F/Q22R/L25R_ was predicted to form one pi–pi interaction with Y489 of SARS-CoV-2-RBD. Moreover, other residues were predicted to form one pi–pi interaction (F20-Y505), two cation–pi interactions (Y21-R403:NH1 and Y21-R403:NH2) and one sigma–pi (Y21-Y505:HD2) interactions between SPB25_K11F_ and SARS-CoV-2-RBD.

### Peptide helicities of designed peptides with predicted binding affinities to SARS-CoV-2-RBD better than or similar to ACE2

**Figure 5:**
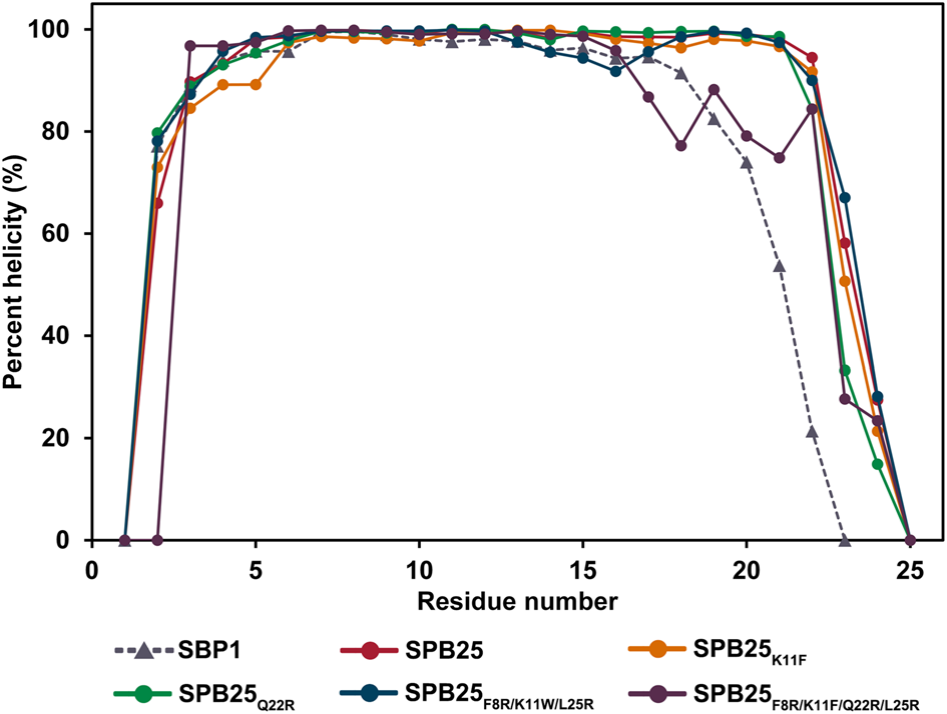
The percent helicities in water of SBP1^36^, SPB25^36^ and designed peptides with predicted binding affinities to SARS-CoV-2-RBD better than or similar to ACE2.

The RMSD plots and the percent helicities of designed peptides in water with predicted binding affinities to SARS-CoV-2-RBD better than or similar to ACE2 are shown in Figure S2 and Fig. 5, respectively. Because of their high flexibilities, the percent helicities of the N terminus and C terminus of each peptide are lower than those of the residues in the middle. Overall, the trends of percent helicities in water of SPB25_K11F_, SPB25_Q22R_, SPB25_F8R/K11W//L25R_ SPB25_F8R/K11F/Q22R/L25R_ and SPB25 are slightly higher than those of SBP1 (the experimentally proven peptide binder of SARS-CoV-2).

## Discussion

COVID-19 pandemic has caused large numbers of cases and deaths globally, and it is caused by SARS-CoV-2 that initially uses its SARS-CoV-2-RBD to bind to ACE2-PD to enter human cells. Therefore, inhibiting the binding between SARS-CoV-2-RBD and ACE2-PD is a promising therapeutic solution for COVID-19. As alternatives to small molecules that are often ineffective in inhibiting large protein binding interfaces^43^, peptides can potentially be used as SARS-CoV-2 inhibitors because their surfaces are larger and they have more functional groups and similar interactions to the native protein–protein interactions than small molecules^20^.

Designed based on residues 21–43 of the α1 helix of ACE2-PD, the 23-mer peptide (SBP1) was experimentally found to bind to SARS-CoV-2-RBD with lower binding affinity than ACE2 and could potentially be used as a SARS-CoV-2 inhibitor^32^. To further enhance the binding affinity of SBP1, our previous study employed computational protein design (Rosetta) and MD (AMBER) to design 25-mer peptide binders of SARS-CoV-2-RBD, based on residues 21–45 of the α1 helix of ACE2-PD (SPB25), by using residues that have not been reported to form favorable interactions with SARS-CoV-2-RBD to increase favorable interactions of these residues and avoid disrupting existing favorable interactions. Five designed peptides such as SPB25_F8N_, SPB25_F8R_, SPB25_L25R_, SPB25_F8N/L25R_ and SPB25_F8R/L25R_ were predicted to bind to SARS-CoV-2-RBD with better binding affinities than SBP1 and SPB25. However, their predicted binding affinities to SARS-CoV-2-RBD are still lower than human ACE2 receptor. The aim of this study is to further increase the binding affinities of 25-mer peptides so that their predicted binding affinities are better than human ACE2 receptor using computational protein design (Rosetta) and MD (AMBER). Using SPB25 as a designed template and reference, our design strategy is to enhance the binding affinity of residues that were previously reported to form favorable interactions between residue 21–45 of ACE2-PD and SARS-CoV-2-RBD^29,37^ and combine the newly designed single mutations with the best designed single mutations (SPB25_F8N_, SPB25_F8R_ and SPB25_L25R_) from our previous study to further increase the binding affinities of designed peptides so that their predicted binding affinities are better than human ACE2 receptor. In this study, designed positions were selected from residues that were previously reported to form favorable interactions with SARS-CoV-2-RBD^29,37^ and their side chains could potentially form favorable interactions upon mutations with SARS-CoV-2-RBD. Q4(24), T7(27), D10(30), K11(31), H14(34), E15(35), E17(37), D18(38), Y21(41) and Q22(42) of SPB25 were selected for design based on our criteria, and they were allowed to be any of standard amino acids except G and P. The total of 156 designed peptides with single mutations were obtained from Rosetta, and the values of ΔG_bind (Rosetta)_ of ten designed peptides are better than that of SPB25 (ΔΔG_bind (Rosetta)_ < 0 REU). These ten designed peptides were selected for MD, and their binding free energies (ΔG_bind (MM-GBSA)_) were calculated by the more accurate MM-GBSA method to determine whether their predicted binding affinities were better than that of SPB25. Our results show that three designed peptides with single mutations such as SPB25_K11F,_ SPB25_K11W_ and SPB25_Q22R_ were predicted to bind to SARS-CoV-2-RBD better than SPB25 with ΔΔG_bind (MM-GBSA)_ of − 11.3 ± 0.7, − 2.9 ± 0.6 and − 15.0 ± 0.6 kcal/mol, respectively. These three designed single mutations were combined with the three best designed single mutations (SPB25_F8N_, SPB25_F8R_ and SPB25_L25R_) from our previous work to construct 11, 12 and 4 designed peptides with double, triple and quadruple mutations using Rosetta (SPB25_F8N/L25R_ and SPB25_F8R/L25R_ were not included in this study because their predicted binding affinities were already reported in our previous study). MD was performed on these designed peptides, and their values of ΔG_bind (MM-GBSA)_ were computed.

In terms of designed peptides with double mutations, SPB25_F8N/K11W_, SPB25_F8R/K11F_, SPB25_F8R/K11W_, SPB25_F8R/Q22R_, SPB25_K11F/L25R_ and SPB25_K11W/L25R_ were predicted to bind to SARS-CoV-2-RBD better than SPB25 with ΔΔG_bind (MM-GBSA)_ of − 6.5 ± 0.6, − 9.4 ± 0.6, − 9.2 ± 0.6, − 3.6 ± 0.5, − 2.8 ± 0.6 and − 4.0 ± 0.6 kcal/mol, respectively. For designed peptides with triple mutations, SPB25_F8N/K11F/L25R_, SPB25_F8N/K11W/L25R_, SPB25_F8R/K11W/L25R_ and SPB25_K11W/Q22R/L25R_ were predicted to bind to SARS-CoV-2-RBD better than SPB25 with ΔΔG_bind (MM-GBSA)_ of − 0.3 ± 0.6, − 1.4 ± 0.6, − 14.7 ± 0.5 and − 7.5 ± 0.6 kcal/mol, respectively. In terms of designed peptides with quadruple mutation, SPB25_F8R/K11F/Q22R/L25R_ and SPB25_F8R/K11W/Q22R/L25R_ were predicted to bind to SARS-CoV-2-RBD better than SPB25 with ΔΔG_bind (MM-GBSA)_ of − 11.9 ± 0.6 and − 7.1 ± 0.6 kcal/mol, respectively. All designed peptides were also predicted to bind to SARS-CoV-2-RBD better than SBP1 (the experimentally proven peptide binder of SARS-CoV-2-RBD), suggesting that they should be able to bind to SARS-CoV-2-RBD better than SBP1, experimentally. Most importantly, three designed peptides (SPB25_Q22R_, SPB25_F8R/K11W/L25R_ and SPB25_F8R/K11F/Q22R/L25R_) were predicted to bind to SARS-CoV-2-RBD better than ACE2 by − 4.1 ± 0.6, − 3.8 ± 0.5 and − 1.0 ± 0.6 kcal/mol, respectively, suggesting that they should bind to SARS-CoV-2-RBD better than ACE2, experimentally. Moreover, one designed peptide (SPB25_K11F_) was predicted to bind to SARS-CoV-2-RBD with relatively similar binding affinity (− 71.6 ± 0.6) to ACE2 (− 71.2 ± 0.4), suggesting that it should bind to SARS-CoV-2-RBD with relatively similar *K*_D_ to ACE2. The ranking of the predicted binding affinities of the designed peptides, SPB25, SBP1 and ACE2 (best to worst) is SPB25_Q22R_ ≈ SPB25_F8R/K11W/L25R_ > SPB25_F8R/K11F/Q22R/L25R_ > SPB25_K11F_ ≈ ACE2 > SPB25 > SBP1. Although ACE2 is markedly larger and has more residues interacting with SARS-CoV-2-RBD, including residues in the α2 helix and the linker of the β3 and β4 antiparallel strands in addition to residues 21–45^6,9^, than our best designed 25-mer peptides, our approach was able to design 25-mer peptides with better predicted binding affinity than ACE2, suggesting the effectiveness of our approach and the high efficacies of our best designed peptides. Moreover, the binding positions and orientations of all designed peptides to SARS-CoV2-RBD are relatively similar to that of residues 21–45 of the α1 helix of ACE2-PD, suggesting that they could potentially disrupt the binding interactions between SARS-CoV2-RBD and ACE2-PD.

SPB25_Q22R_ is the most promising designed peptide because its predicted binding affinity is better than ACE2, SPB25, SBP1 and all designed peptides. This result is supported by the fact that its total numbers of predicted hydrogen bonds (involving E3, Q4, D10, H14, E15, E17, Y21, R22, S23, S24 and L25) and pi interactions (involving K11, H14, Y21 and R22) are higher than those of SPB25, SBP1 and ACE2. The per-residue free energy decomposition results suggest Q4, T7, F8, K11, H14, E17, Y21 and R22 as important binding residues. Additionally, the Q22R mutation was predicted to cause substantial favorable increase in the total energy contribution of this residue and the total energy contributions of other residues such as Q4, F8, H14, E17, and Y21 as compared to those of SPB25 and ACE2.

SPB25_F8R/K11W/L25R_ was predicted to bind better to SARS-CoV2-RBD than SBP1, SPB25 and ACE2. This result is supported by the fact that its total numbers of predicted hydrogen bonds (involving I1, E3, Q4, R8, D10, H14, E17, Y21, Q22, S24 and R25) and pi interactions (involving W11, R8 and Y21) are higher than those of SBP1 and ACE2, and the number of predicted strong hydrogen bonds of SPB25_F8R/K11W/L25R_ is higher than that of SBP1, SPB25 and ACE2. The predicted binding affinity of SPB25_F8R/K11W/L25R_ is lower than SPB25_Q22R_, and this result is supported by the fact that its total numbers of predicted hydrogen bonds and pi interactions are lower than those of SPB25_Q22R_. The results from per-residue free energy decomposition suggest E3, Q4, T7, R8, D10, W11, H14, E17, Y21, Q22, S24 and R25 as important binding residues. Furthermore, the F8R/K11W/L25R mutation was predicted to cause substantial increase in the total energy contribution of residue 8 and 25 as well as other residues such as E3, Q4, H14, E17, Y21 and S24 as compared to those of SPB25 and ACE2.

The predicted binding affinity of SPB25_F8R/K11F/Q22R/L25R_ is better than those of SBP1, SPB25 and ACE2. This finding is supported by the fact that the numbers of predicted hydrogen bonds (involving Q4, R8, D10, H14, E17, D18, Y21, F20, S24 and R25) and pi interactions (involving R8, F11, H14, F20 and Y21) of SPB25_F8R/K11F/Q22R/L25R_ are higher than those of SBP1, SPB25 and ACE2. Additionally, the predicted numbers of strong and medium hydrogen bonds (involving Q4, R8, H14, E17, and Y21) of SPB25 _F8R/K11F/Q22R/L25R_ are higher than those of ACE2, SPB25 and SBP1. The results from per-residue free energy decomposition suggest E3, Q4, T7, R8, D10, F11, H14, E17, F20, Y21, S24 and R25 as important binding residues. Moreover, the F8R/K11F/Q22R/L25R mutation was predicted to cause the increase in the total energy contribution of residue 8 and 25 and other residues such as Q4, H14, E17, F20, Y21 and S24 as compared to those of SPB25 and ACE2. However, this quadruple mutation decreases the total energy contribution of residue 22 as compared to those of SPB25 probably because R22 of SPB25 _F8R/K11F/Q22R/L25R_ causes a decrease in favorable electrostatic interaction as well as an increase in repulsive interaction between R22 and R25. As a result, its predicted binding affinity is lower than SPB25_Q22R_ and SPB25_F8R/K11W/L25R_.

The binding affinity of SPB25_K11F_ was predicted to be better than those of SBP1, SPB25 and relatively similar to ACE2. The enhanced binding affinity of SPB25_K11F_ is probably caused by the increase in the total numbers of predicted hydrogen bonds (involving E2, Q4, D10, H14, E17, D18, Y21, Q22, S23, S24 and L25) and pi interactions (involving F11, F20 and Y21) of SPB25_K11F_ as compared to those of SBP1, SPB25 and ACE2. SPB25_K11F_ has the worst predicted binding affinity among the four best designed peptides, and this finding is supported by the fact that its total numbers of predicted strong, medium and weak hydrogen bonds as well as pi interactions are the lowest among these four best designed peptides. The results from per-residue free energy decomposition suggest Q4, T7, F8, D10, F11, H14, E17, F20, Y21, Q22 and S24 as important binding residues. Moreover, the K11F mutation caused significant increase in the total energy contributions of other residues such as F8, E17, F20, Y21 and S24 as compared to those of SPB25 and ACE2.

In terms of peptide helicities, the trends of percent helicities in water of SPB25_Q22R_, SPB25_K11F_, SPB25_F8R/K11W/L25R_ and SPB25 _F8R/K11F/Q22R/L25R_ are slightly higher than that of SBP1. These results suggest that their stabilities in water may be slightly better than that of SBP1 (the experimentally proven binder of SARS-CoV-2-RBD), and these designed peptides should be stable enough to be used as peptide binders of SARS-CoV-2.

Employing computational protein design and MD, we designed three 25-mer peptides (SPB25_Q22R_, SPB25_F8R/K11W/L25R_ and SPB25_F8R/K11F/Q22R/L25R_) and one 25-mer peptide (SPB25_K11F_) with predicted binding affinities better than and similar to that of human ACE2 receptor, respectively. Although their sizes are markedly smaller than human ACE2 receptor, they were predicted to bind to SARS-CoV-2-RBD with better or similar binding affinities, suggesting their high efficacies. These four designed peptides are promising candidates that could potentially be employed as inhibitors to prevent the binding of SARS-CoV-2-RBD and ACE2. One potential application is to use these designed peptides as inhaled therapeutics for topical lung delivery to prevent the binding of SARS-CoV-2-RBD and ACE2 in the lung^44^. Moreover, these 25-mer peptide binders are approximately 40-fold smaller than a full antibody molecule; therefore, they have roughly 40-fold more potential neutralizing sites than a full antibody molecule at the same equal mass, thereby enhancing their potential efficacies. Furthermore, since they do not require expression in mammalian cells for proper folding like antibodies, the cost of scale-up and increase production volumes of these peptides should be lower than those of antibodies. Their small sizes should also allow them to be formulated in a gel for nasal application as well as to be delivered to the respiratory system as a dry powder or by nebulization^15^.

In conclusion, we developed an approach to design 25-mer peptide binders of SARS-CoV-2 with predicted binding affinities better than human ACE2 receptors, using computational protein design and MD. Employing SPB25 (residue 21–45 of ACE2-PD) as a designed template, our design strategy is to enhance the binding affinity of residues that were previously reported to form favorable interactions between residue 21–45 of ACE2-PD and SARS-CoV-2-RBD and combine the newly designed single mutations with the best designed single mutations (SPB25_F8N_, SPB25_F8R_ and SPB25_L25R_) from our previous study to further increase the binding affinities of designed peptides so that their predicted binding affinities are better than human ACE2 receptor. Using this strategy, we designed three 25-mer peptides (SPB25_Q22R_, SPB25_F8R/K11W/L25R_ and SPB25_F8R/K11F/Q22R/L25R_) and one 25-mer peptide (SPB25_K11F_) with predicted binding affinities to SARS-CoV-2-RBD, by the MM-GBSA method, better than and similar to human ACE2 receptor, respectively. Moreover, their predicted helicities in water are slightly higher than SBP1 (the experimentally proven 23-mer peptide binder of SARS-CoV-2-RBD), suggesting that their stabilities may be slightly better than SBP1. These four peptides are promising candidates as SARS-CoV-2 inhibitors.

## Methods

### Structure preparation

The 25-mer peptide of SPB25 (21 IEEQAKTFLDKFNHEAEDLFYQSSL 45) bound to SARS-CoV-2-RBD complex was obtained from our previous work^36^ and it was constructed from the crystal structure of α1 helix of ACE2 peptidase domain (ACE2-PD) bound to SARS-COV-2-RBD (PDB ID: 6M0J^37^). The complex was protonated at the physiological pH (pH 7.4) using H^++^ server^45^. The LEaP module of AMBER18^46^ was used to build the final structure of the complex.

### Computational protein design

The structure of SPB25/SARS-CoV-2-RBD complex was employed as a template to design the SARS-CoV-2-RBD peptide binders using Rosetta. Our design strategy is to increase the binding affinity of residues that were previously reported to form favorable interactions between residue 21–45 of ACE2 and SARS-CoV-2-RBD^29,37^ and further combine the newly designed single mutations with the best designed single mutations from our previous study to further increase the binding affinities of designed peptides so that their predicted binding affinities are better than ACE2. Obtained from our previous study, these best designed mutations were designed from the residues that have not been reported to form favorable interactions with SARS-CoV-2-RBD to increase favorable interactions of these residues and avoid disrupting existing favorable interactions. In this study, designed positions were selected from residues that were previously reported to form favorable interactions with SARS-CoV-2-RBD^29,37^ and their side chains could potentially form favorable interactions upon mutations with SARS-CoV-2-RBD. The structure of designed residues were designed, repacked and minimized using the CoupledMoves protocol^47,48^ in RosettaDesign module of Rosetta3.11^49^ with beta_nov16 energy function. The designed positions were allowed to be any of standard amino acids except G and P, and the neighboring residues within 10 Å of designed position were also repacked and minimized. 400 independent runs were performed, and the total of 400 conformation of designed sequences were obtained for each design (some sequences may have multiple conformations). The binding free energy [ΔG_bind (Rosetta)_] of each designed conformation was calculated in Rosetta Energy Unit (REU) using Interface Analyzer^50,51^ module of Rosetta3.11. ΔΔG_bind (Rosetta)_ upon mutation was computed by subtracting the values of ΔG_bind (Rosetta)_ between the designed conformation and SPB25 conformation. The designed conformations with the best binding free energy and ΔΔG_bind (Rosetta)_ < 0 REU of each design position were selected for MD simulations to validate their predicted binding affinities by the MM-GBSA method^39–41^.

### MD simulations and analyses

Using protein.ff14SB^52^ and GLYCAM06j-1 force field parameters^53^ in AMBER18^46^, the structures of designed peptides/SARS-CoV-2-RBD complexes were constructed in isomeric truncated octahedral boxes of TIP3P water molecules with the buffer distance of 13 Å. Each system was minimized using the five-step procedure^42,54–59^. All minimization steps include 2500 steps of steepest descent and 2500 steps of conjugate gradient with different restraints on the proteins to remove unfavorable interactions. In the first step, the hydrogen atoms and water molecules were minimized, while the heavy atoms of proteins were restrained with a force constant of 10 kcal/(mol Å^2^). The backbones of the proteins were then restrained with the force constants of 10, 5 and 1 kcal/(mol Å^2^) in the second, third and fourth steps of minimizations, respectively. Finally, no restraining force was applied in the system.

All systems were simulated with the periodic boundary condition, using the GPU (CUDA) version of PMEMD module^60–62^. The SHAKE algorithm^63^ was employed to constrain all bonds involving hydrogen atoms, allowing the time step of 0.002 ps. The Langevin dynamics technique was applied to control the temperatures of all systems with a collision frequency of 1.0 ps^−1^_._ All systems were heated from 0 K to the physiological temperature of 310 K in the NVT ensemble for 200 ps, and a force constant of 10 kcal/(mol Å^2^) was applied to restrain the backbones of the proteins. All systems were then equilibrated without restraint at 310 K in the NVT ensemble for 300 ps. Finally, they were subsequently simulated at 310 K and 1 atm in the NPT ensemble for 100 ns.

To analyze the stability of each system, the Root Mean Square Deviation (RMSD) values with respect to the minimized structure were calculated. The 80–100 ns trajectories of all systems with stable RMSD values were chosen for further analyses. To predict the binding affinities between designed peptides and SARS-CoV-2-RBD, the MM-GBSA method was used to calculate the total binding free energies [ΔG_bind (MM-GBSA)_] of all systems. The designed peptides with better predicted binding affinity than ACE2 were further analyzed in terms of per-residue free energy decomposition and binding interactions. Hydrogen bond occupations were computed to analyze hydrogen bond interactions. In this study, a hydrogen bond was considered to occur if the following criteria were met: (1) a proton donor–acceptor distance ≤ 3.5 Å and (2) a donor-H-acceptor bond angle ≥ 120°^42,54,55,64^. Hydrogen bond occupations were defined into four levels: (1) strong hydrogen bonds (hydrogen bond occupations > 75%), (2) medium hydrogen bonds (75% ≥ hydrogen bond occupations > 50%), (3) weak hydrogen bond interactions (50% ≥ hydrogen bond occupations > 25%) and (4) very weak hydrogen bond interactions (25% ≥ hydrogen bond occupations > 5%)^42,55,56^. To compute peptide helicities, Define Secondary Structure of Protein (DSSP) was employed. Percent helicity was calculated from the summation of the percentage of α-, 3_10_- and pi-helix structures^65^.

## Supplementary Information

Supplementary Information 1.

## Data Availability

All data generated or analyzed during this study are included in this published article (and its Supplementary Information files).
