# Supplementary material for: Computational design of SARS-CoV-2 peptide binders with better predicted binding affinities than human ACE2 receptor
Source: Sci Rep. 2021 Aug 2;11:15650. doi: 10.1038/s41598-021-94873-3 (PMC8329052; doi:10.1038/s41598-021-94873-3)
Supplement: Supplementary file 1 — Supplementary Information 1. [file 41598_2021_94873_MOESM1_ESM.pdf]

Computational design of SARS-CoV-2 peptide binders with better predicted binding affinities than human ACE2 receptor

Thassanai Sitthiyotha[a], Surasak Chunsrivirot[a,b,]*

[a]Structural and Computational Biology Research Unit, Department of Biochemistry, Faculty of Science, Chulalongkorn University, Pathumwan, Bangkok 10330, Thailand
[b]Department of Biochemistry, Faculty of Science, Chulalongkorn University, Pathumwan, Bangkok 10330, Thailand

[*]Surasak Chunsrivirot
Email: surasak.ch@chula.ac.th

Contents

Figure S1. RMSD plots of designed peptides in complex with SARS-CoV-2-RBD, S2-S3

Figure S2. RMSD plots of designed peptides with predicted binding affinities to SARS-CoV-2-RBD better than or similar to ACE2 in water, S4

Table S1. The binding free energies of ACE2, SBP1, SPB25 and all designed peptides to SARS-CoV-2-RBD as calculated by Rosetta and MM-GBSA method, S5-S11

Table S2: Hydrogen bond occupations of ACE2, SBP1, SPB25 and designed peptides binding to SARS-CoV-2-RBD, S12-S15

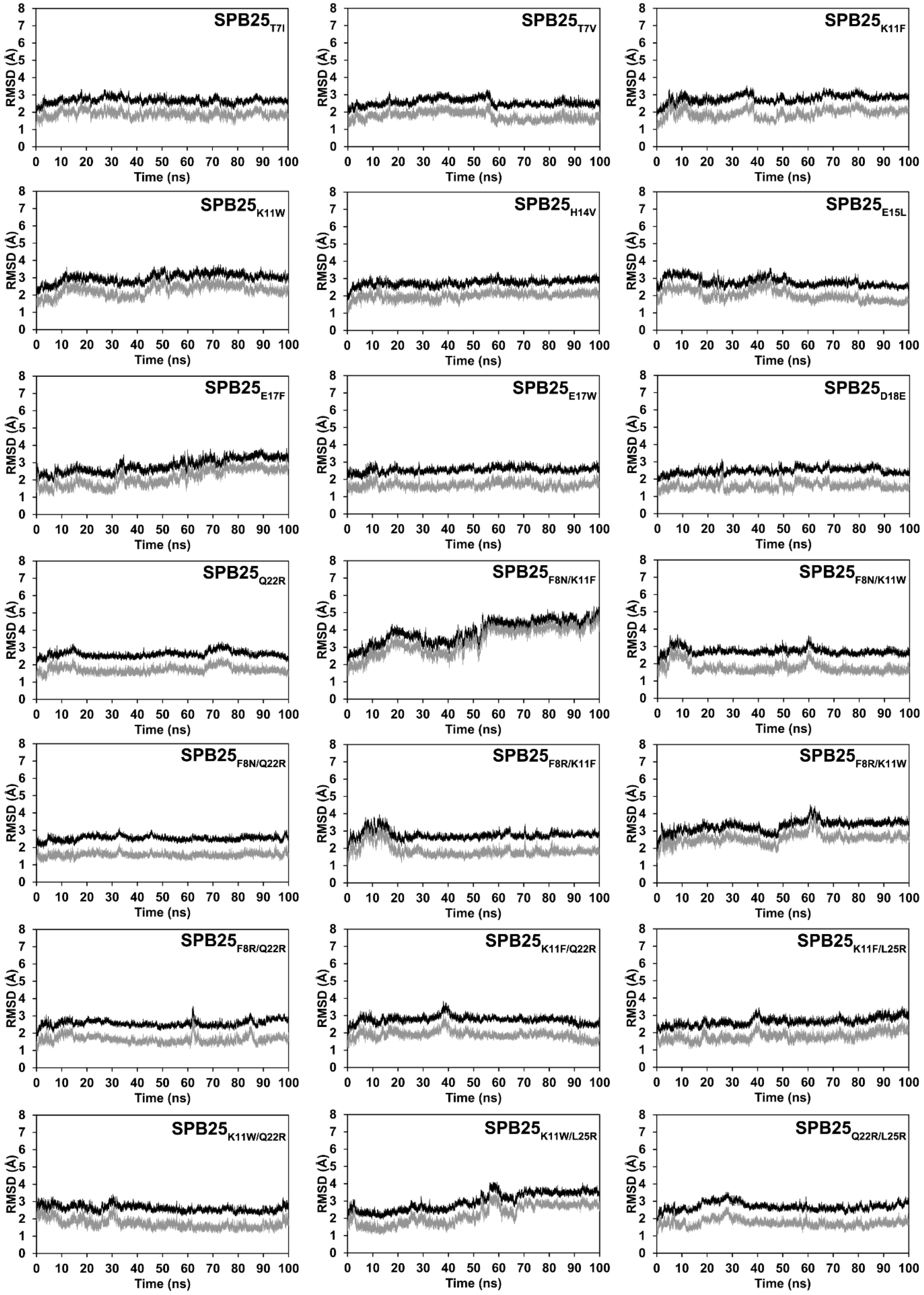
$SPB25_{T7I}$
$SPB25_{T7V}$
$SPB25_{K11F}$
$SPB25_{K11W}$
$SPB25_{H14V}$
$SPB25_{E15L}$
$SPB25_{E17F}$
$SPB25_{E17W}$
$SPB25_{D18E}$
$SPB25_{Q22R}$
$SPB25_{F8N/K11F}$
$SPB25_{F8N/K11W}$
$SPB25_{F8N/Q22R}$
$SPB25_{F8R/K11F}$
$SPB25_{F8R/K11W}$
$SPB25_{F8R/Q22R}$
$SPB25_{K11F/Q22R}$
$SPB25_{K11F/L25R}$
$SPB25_{K11W/Q22R}$
$SPB25_{K11W/L25R}$
$SPB25_{Q22R/L25R}$
RMSD (Å)
Time (ns)

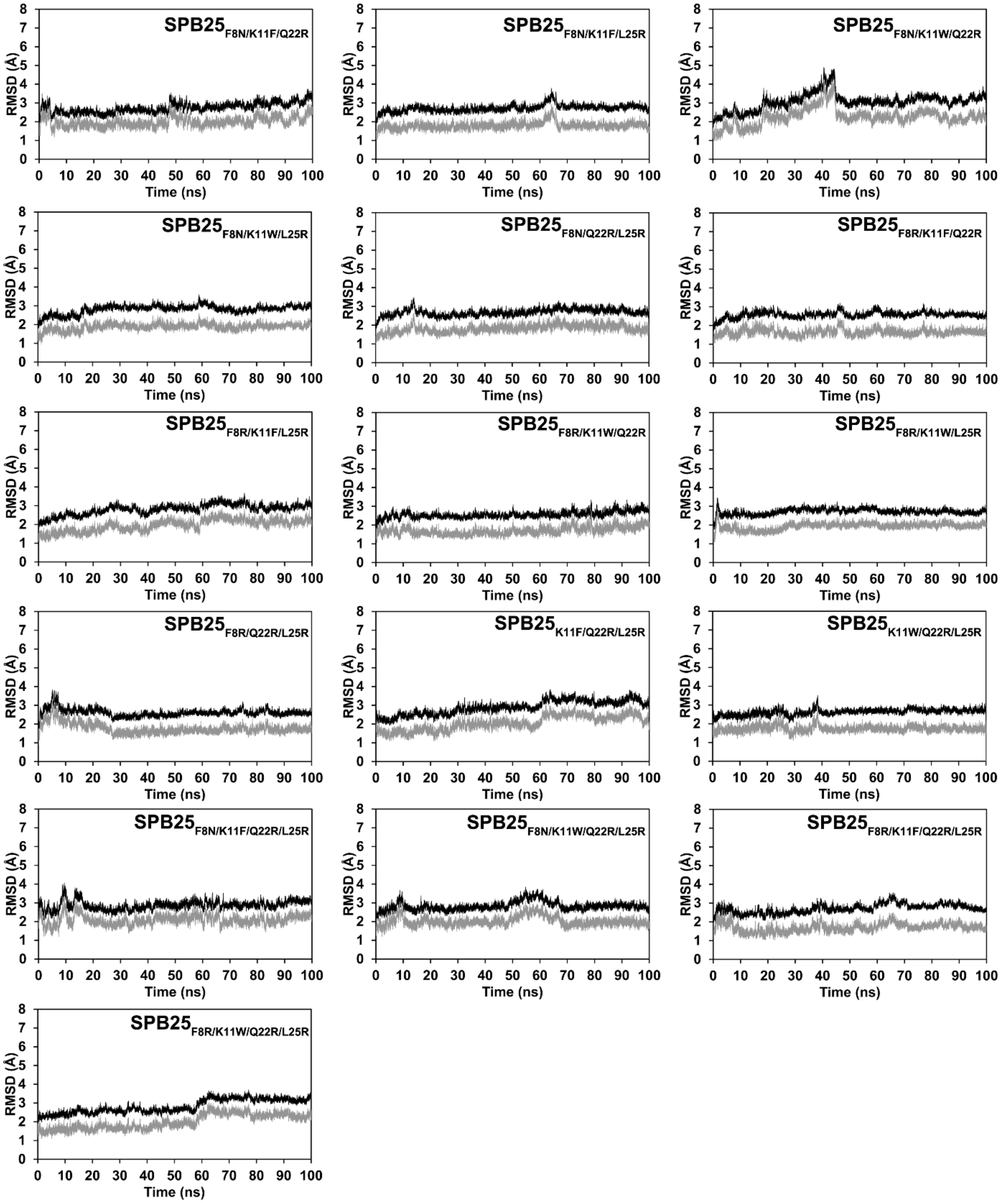

Figure S1. RMSD plots of designed peptides in complex with SARS-CoV-2-RBD. The RMSD values of all atoms and backbone atoms are shown in black and grey, respectively.

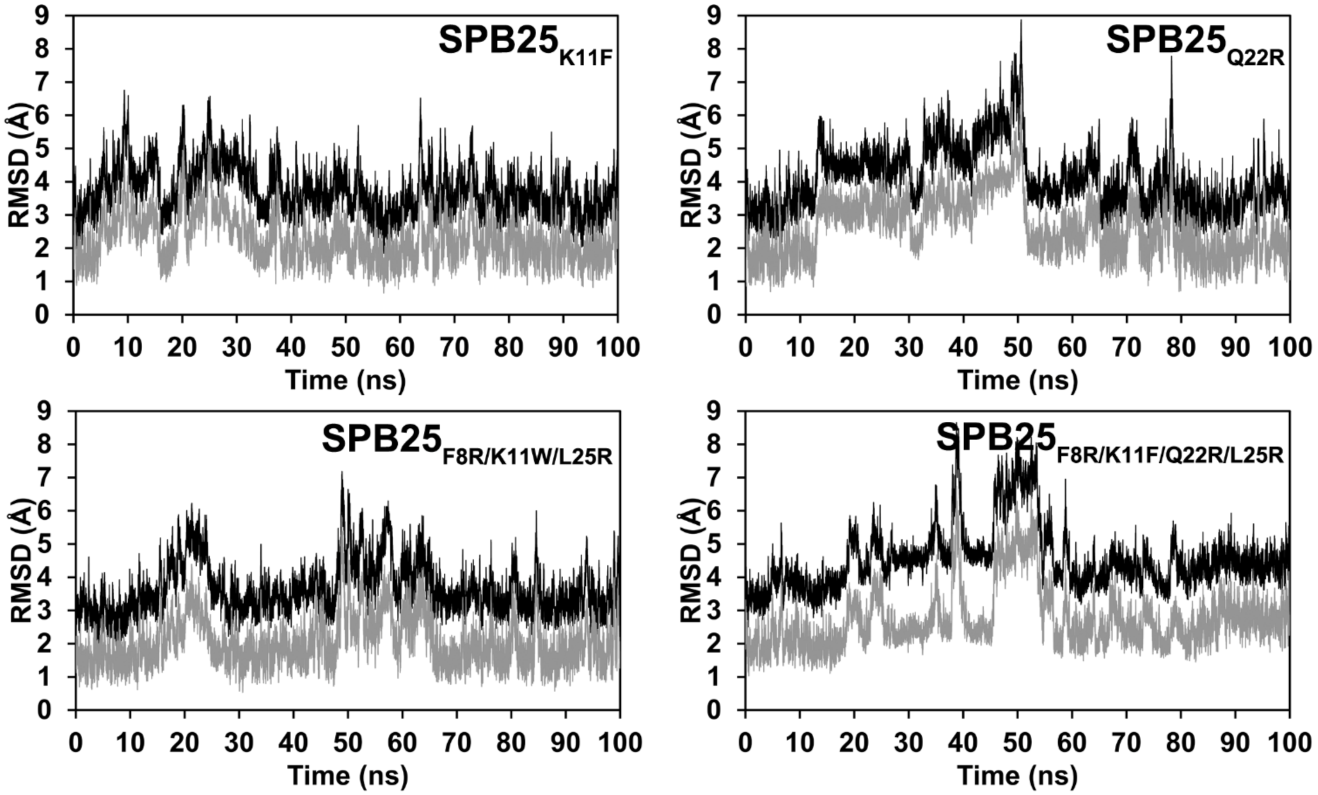

Figure S2. RMSD plots of designed peptides with predicted binding affinities to SARS-CoV-2-RBD better than or similar to ACE2 in water. The RMSD values of all atoms and backbone atoms are shown in black and grey respectively.

Table S1. The binding free energies of ACE2, SBP1, SPB25 and all designed peptides to SARS-CoV-2-RBD as calculated by Rosetta and MM-GBSA method.

System	$\Delta\Delta G_{bind\ (Rosetta)}$[a] (REU)	$\Delta G_{bind\ (MM\text{-}GBSA)}$ (kcal/mol)	$\Delta\Delta G_{bind\ (MM\text{-}GBSA)}$[b] (kcal/mol)
ACE2	-	-71.2 ± 0.4	-10.9 ± 0.6
SBP1	-	-55.1 ± 0.4	5.2 ± 0.6
SPB25	0.0	-60.3 ± 0.4	0.0 ± 0.6
$SPB25_{Q4A}$	3.1	-	-
$SPB25_{Q4D}$	8.4	-	-
$SPB25_{Q4E}$	2.0	-	-
$SPB25_{Q4H}$	3.4	-	-
$SPB25_{Q4I}$	0.8	-	-
$SPB25_{Q4L}$	0.6	-	-
$SPB25_{Q4M}$	5.5	-	-
$SPB25_{Q4R}$	3.1	-	-
$SPB25_{Q4S}$	3.9	-	-
$SPB25_{Q4T}$	2.6	-	-
$SPB25_{Q4V}$	1.4	-	-
$SPB25_{Q4W}$	1.1	-	-
$SPB25_{T7A}$	2.5	-	-
$SPB25_{T7D}$	7.1	-	-
$SPB25_{T7F}$	1.6	-	-
$SPB25_{T7H}$	4.3	-	-
$SPB25_{T7I}$	-0.3	-59.2 ± 0.3	1.1 ± 0.5
$SPB25_{T7L}$	0.1	-	-
$SPB25_{T7M}$	2.3	-	-
$SPB25_{T7N}$	7.1	-	-
$SPB25_{T7Q}$	6.4	-	-
$SPB25_{T7R}$	4.4	-	-
$SPB25_{T7S}$	8.1	-	-

System	$\Delta\Delta G_{bind\ (Rosetta)}$[a] (REU)	$\Delta G_{bind\ (MM\text{-}GBSA)}$ (kcal/mol)	$\Delta\Delta G_{bind\ (MM\text{-}GBSA)}$[b] (kcal/mol)
$SPB25_{T7V}$	-0.4	-50.9 ± 0.3	9.4 ± 0.5
$SPB25_{T7W}$	2.7	-	-
$SPB25_{T7Y}$	2.8	-	-
$SPB25_{D10A}$	3.5	-	-
$SPB25_{D10C}$	3.9	-	-
$SPB25_{D10E}$	2.5	-	-
$SPB25_{D10H}$	3.6	-	-
$SPB25_{D10I}$	4.9	-	-
$SPB25_{D10K}$	5.7	-	-
$SPB25_{D10L}$	6.6	-	-
$SPB25_{D10M}$	6.9	-	-
$SPB25_{D10N}$	4.8	-	-
$SPB25_{D10Q}$	5.2	-	-
$SPB25_{D10R}$	5.4	-	-
$SPB25_{D10S}$	2.1	-	-
$SPB25_{D10T}$	7.3	-	-
$SPB25_{D10V}$	4.8	-	-
$SPB25_{D10W}$	3.1	-	-
$SPB25_{D10Y}$	4.9	-	-
$SPB25_{K11A}$	1.9	-	-
$SPB25_{K11E}$	6.7	-	-
$SPB25_{K11F}$	-0.4	-71.6 ± 0.6	-11.3 ± 0.7
$SPB25_{K11H}$	2.1	-	-
$SPB25_{K11I}$	2.2	-	-
$SPB25_{K11M}$	0.8	-	-
$SPB25_{K11N}$	3.3	-	-
$SPB25_{K11Q}$	2.3	-	-
$SPB25_{K11R}$	2.4	-	-
$SPB25_{K11S}$	4.7	-	-

System	$\Delta\Delta G_{bind\ (Rosetta)}$[a] (REU)	$\Delta G_{bind\ (MM\text{-}GBSA)}$ (kcal/mol)	$\Delta\Delta G_{bind\ (MM\text{-}GBSA)}$[b] (kcal/mol)
$SPB25_{K11T}$	8.4	-	-
$SPB25_{K11W}$	-2.2	-63.2 ± 0.4	-2.9 ± 0.6
$SPB25_{K11Y}$	0.3	-	-
$SPB25_{H14A}$	3.0	-	-
$SPB25_{H14C}$	2.7	-	-
$SPB25_{H14D}$	4.0	-	-
$SPB25_{H14E}$	4.6	-	-
$SPB25_{H14F}$	5.3	-	-
$SPB25_{H14I}$	2.4	-	-
$SPB25_{H14K}$	5.2	-	-
$SPB25_{H14L}$	6.6	-	-
$SPB25_{H14M}$	5.1	-	-
$SPB25_{H14N}$	3.9	-	-
$SPB25_{H14Q}$	6.2	-	-
$SPB25_{H14R}$	5.8	-	-
$SPB25_{H14S}$	3.8	-	-
$SPB25_{H14T}$	5.1	-	-
$SPB25_{H14V}$	-0.1	-58.2 ± 0.5	2.1 ± 0.6
$SPB25_{H14W}$	4.9	-	-
$SPB25_{H14Y}$	2.5	-	-
$SPB25_{E15A}$	0.7	-	-
$SPB25_{E15D}$	0.8	-	-
$SPB25_{E15F}$	1.5	-	-
$SPB25_{E15H}$	1.7	-	-
$SPB25_{E15I}$	1.6	-	-
$SPB25_{E15K}$	2.0	-	-
$SPB25_{E15L}$	-0.9	-51.7 ± 0.4	8.6 ± 0.6
$SPB25_{E15M}$	1.2	-	-
$SPB25_{E15N}$	1.3	-	-

System	$\Delta\Delta G_{bind\ (Rosetta)}$[a] (REU)	$\Delta G_{bind\ (MM\text{-}GBSA)}$ (kcal/mol)	$\Delta\Delta G_{bind\ (MM\text{-}GBSA)}$[b] (kcal/mol)
$SPB25_{E15Q}$	2.1	-	-
$SPB25_{E15R}$	1.6	-	-
$SPB25_{E15S}$	1.0	-	-
$SPB25_{E15T}$	1.4	-	-
$SPB25_{E15V}$	1.9	-	-
$SPB25_{E15W}$	0.8	-	-
$SPB25_{E15Y}$	1.6	-	-
$SPB25_{E17A}$	0.5	-	-
$SPB25_{E17C}$	3.8	-	-
$SPB25_{E17F}$	-0.9	-47.7 ± 0.4	12.6 ± 0.6
$SPB25_{E17H}$	1.9	-	-
$SPB25_{E17I}$	2.1	-	-
$SPB25_{E17K}$	2.6	-	-
$SPB25_{E17L}$	1.3	-	-
$SPB25_{E17M}$	3.1	-	-
$SPB25_{E17N}$	6.2	-	-
$SPB25_{E17Q}$	2.6	-	-
$SPB25_{E17R}$	2.5	-	-
$SPB25_{E17S}$	3.1	-	-
$SPB25_{E17T}$	2.3	-	-
$SPB25_{E17W}$	-3.1	-57.8 ± 0.5	2.5 ± 0.6
$SPB25_{E17Y}$	0.6	-	-
$SPB25_{D18A}$	7.1	-	-
$SPB25_{D18E}$	-0.5	-55.4 ± 0.4	4.9 ± 0.6
$SPB25_{D18F}$	4.3	-	-
$SPB25_{D18H}$	2.5	-	-
$SPB25_{D18I}$	2.0	-	-
$SPB25_{D18L}$	1.1	-	-
$SPB25_{D18M}$	4.5	-	-

System	$\Delta\Delta G_{bind\ (Rosetta)}$[a] (REU)	$\Delta G_{bind\ (MM\text{-}GBSA)}$ (kcal/mol)	$\Delta\Delta G_{bind\ (MM\text{-}GBSA)}$[b] (kcal/mol)
$SPB25_{D18N}$	4.3	-	-
$SPB25_{D18Q}$	3.4	-	-
$SPB25_{D18R}$	7.2	-	-
$SPB25_{D18S}$	7.5	-	-
$SPB25_{D18T}$	4.0	-	-
$SPB25_{D18V}$	4.6	-	-
$SPB25_{D18W}$	3.1	-	-
$SPB25_{D18Y}$	4.4	-	-
$SPB25_{Y21A}$	6.0	-	-
$SPB25_{Y21D}$	9.5	-	-
$SPB25_{Y21E}$	5.2	-	-
$SPB25_{Y21F}$	3.2	-	-
$SPB25_{Y21H}$	5.9	-	-
$SPB25_{Y21I}$	7.0	-	-
$SPB25_{Y21K}$	7.4	-	-
$SPB25_{Y21L}$	4.4	-	-
$SPB25_{Y21N}$	7.9	-	-
$SPB25_{Y21Q}$	6.6	-	-
$SPB25_{Y21R}$	5.2	-	-
$SPB25_{Y21S}$	7.3	-	-
$SPB25_{Y21T}$	5.1	-	-
$SPB25_{Y21V}$	6.2	-	-
$SPB25_{Y21W}$	4.0	-	-
$SPB25_{Q22A}$	2.4	-	-
$SPB25_{Q22D}$	4.3	-	-
$SPB25_{Q22E}$	2.8	-	-
$SPB25_{Q22F}$	0.4	-	-
$SPB25_{Q22H}$	3.7	-	-
$SPB25_{Q22I}$	2.0	-	-

System	$\Delta\Delta G_{bind\ (Rosetta)}$[a] (REU)	$\Delta G_{bind\ (MM\text{-}GBSA)}$ (kcal/mol)	$\Delta\Delta G_{bind\ (MM\text{-}GBSA)}$[b] (kcal/mol)
$SPB25_{Q22K}$	3.4	-	-
$SPB25_{Q22L}$	0.7	-	-
$SPB25_{Q22M}$	2.7	-	-
$SPB25_{Q22N}$	1.5	-	-
$SPB25_{Q22R}$	-0.4	-75.3 ± 0.5	-15.0 ± 0.6
$SPB25_{Q22S}$	3.5	-	-
$SPB25_{Q22T}$	4.4	-	-
$SPB25_{Q22V}$	2.1	-	-
$SPB25_{Q22W}$	2.6	-	-
$SPB25_{F8N/K11F}$	-3.4	-56.8 ± 0.4	3.5 ± 0.6
$SPB25_{F8N/K11W}$	-6.6	-66.8 ± 0.5	-6.5 ± 0.6
$SPB25_{F8N/Q22R}$	-3.0	-58.0 ± 0.3	2.3 ± 0.5
$SPB25_{F8R/K11F}$	-4.9	-69.7 ± 0.5	-9.4 ± 0.6
$SPB25_{F8R/K11W}$	-5.6	-69.5 ± 0.4	-9.2 ± 0.6
$SPB25_{F8R/Q22R}$	-1.5	-63.9 ± 0.3	-3.6 ± 0.5
$SPB25_{K11F/Q22R}$	-3.9	-53.7 ± 0.6	6.6 ± 0.7
$SPB25_{K11F/L25R}$	-3.6	-63.1 ± 0.5	-2.8 ± 0.6
$SPB25_{K11W/Q22R}$	-4.0	-44.0 ± 0.4	16.3 ± 0.6
$SPB25_{K11W/L25R}$	-3.6	-64.3 ± 0.4	-4.0 ± 0.6
$SPB25_{Q22R/L25R}$	-1.8	-47.7 ± 0.4	12.6 ± 0.6
$SPB25_{F8N/K11F/Q22R}$	-6.3	-48.5 ± 0.6	11.8 ± 0.7
$SPB25_{F8N/K11F/L25R}$	-3.9	-60.6 ± 0.5	-0.3 ± 0.6
$SPB25_{F8N/K11W/Q22R}$	-5.8	-56.5 ± 0.5	3.8 ± 0.6
$SPB25_{F8N/K11W/L25R}$	-3.8	-61.7 ± 0.4	-1.4 ± 0.6
$SPB25_{F8N/Q22R/L25R}$	-2.0	-58.1 ± 0.4	2.2 ± 0.6
$SPB25_{F8R/K11F/Q22R}$	-4.5	-59.3 ± 0.4	1.0 ± 0.6
$SPB25_{F8R/K11F/L25R}$	-2.4	-58.1 ± 0.4	2.2 ± 0.6
$SPB25_{F8R/K11W/Q22R}$	-3.6	-52.9 ± 0.4	7.4 ± 0.6
$SPB25_{F8R/K11W/L25R}$	-5.1	-75.0 ± 0.3	-14.7 ± 0.5

System	$\Delta\Delta G_{bind\ (Rosetta)}$[a] (REU)	$\Delta G_{bind\ (MM\text{-}GBSA)}$ (kcal/mol)	$\Delta\Delta G_{bind\ (MM\text{-}GBSA)}$[b] (kcal/mol)
$SPB25_{F8R/Q22R/L25R}$	-0.2	-60.2 ± 0.4	0.1 ± 0.6
$SPB25_{K11F/Q22R/L25R}$	-2.5	-53.7 ± 0.4	6.6 ± 0.6
$SPB25_{K11W/Q22R/L25R}$	-1.2	-67.8 ± 0.5	-7.5 ± 0.6
$SPB25_{F8N/K11F/Q22R/L25R}$	-6.8	-58.7 ± 0.6	1.6 ± 0.7
$SPB25_{F8N/K11W/Q22R/L25R}$	-4.0	-60.2 ± 0.4	0.1 ± 0.6
$SPB25_{F8R/K11F/Q22R/L25R}$	-2.8	-72.2 ± 0.4	-11.9 ± 0.6
$SPB25_{F8R/K11W/Q22R/L25R}$	-3.9	-67.4 ± 0.4	-7.1 ± 0.6

[a] The difference between $\Delta G_{bind\ (Rosetta)}$ of a system and that of SPB25.
[b] The difference between $\Delta G_{bind\ (MM\text{-}GBSA)}$ of a system and that of SPB25.

Table S2: Hydrogen bond occupations of ACE2, SBP1, SPB25 and designed peptides binding to SARS-CoV-2-RBD.

Acceptor[a]	DonorH[a]	Donor[a]	Hydrogen bond occupancy (%)						
			ACE2[36]	SBP1[36]	SPB25[36]	$SPB25_{K11F}$	$SPB25_{Q22R}$	$SPB25_{F8R/K11W/L25R}$	$SPB25_{F8R/K11F/Q22R/L25R}$
A475@O	S(19)@H	S(19)@N	6.5	-	-	-	-	-	-
A475@O	S(19)@HG	S(19)@OG	59.3	-	-	-	-	-	-
S477@OG	I1(21)@H2	I1(21)@N	-	-	-	-	-	5.2	-
S477@OG	I1(21)@H3	I1(21)@N	-	-	-	-	-	5.2	-
E2(22)@O	N487@HD21	N487@ND2	-	-	-	17.4	-	-	-
E2(22)@OE1	S477@H	S477@N	-	-	-	33.1	-	-	-
E2(22)@OE1	S477@HG	S477@OG	-	-	-	16.6	-	-	-
E2(22)@OE1	T478@H	T478@N	-	-	-	6.5	-	-	-
E2(22)@OE1	T478@HG1	T478@OG1	-	-	-	8.2	-	-	-
E2(22)@OE2	S477@H	S477@N	-	-	-	25.7	-	-	-
E2(22)@OE2	S477@HG	S477@OG	-	0.1	-	13.0	-	-	-
E2(22)@OE2	T478@H	T478@N	-	-	-	15.7	-	-	-
E2(22)@OE2	T478@HG1	T478@OG1	-	-	-	15.9	-	-	-
E3(23)@OE1	Y473@HH	Y473@OH	-	-	-	-	7.2	-	-
E3(23)@OE2	S477@H	S477@N	-	-	0.3	-	-	25.7	0.1
E3(23)@OE2	Y473@HH	Y473@OH	0.1	-	-	-	7.4	-	-
A475@O	Q4(24)@HE21	Q4(24)@NE2	-	5.7	11.3	18.8	8.3	3.0	-
A475@O	Q4(24)@HE22	Q4(24)@NE2	5.6	-	1.4	7.6	-	-	-
N487@ND2	Q4(24)@HE21	Q4(24)@NE2	-	0.8	0.4	1.8	6.1	0.4	-
N487@OD1	Q4(24)@H	Q4(24)@N	-	-	-	5.7	-	-	-
N487@OD1	Q4(24)@HE21	Q4(24)@NE2	-	0.8	0.7	7.1	24.4	0.3	-
Q4(24)@NE2	S477@H	S477@N	0.5	20.7	4.2	0.1	2.9	14.8	56.9
Q4(24)@NE2	T478@HG1	T478@OG1	-	4.8	1.2	-	3.3	11.9	0.8
Q4(24)@OE1	N487@HD21	N487@ND2	54.1	7.4	11.3	5.7	2.6	4.1	18.8
Q4(24)@OE1	N487@HD22	N487@ND2	-	4.5	1.6	0.3	1.6	5.1	-
Q4(24)@OE1	T478@H	T478@N	-	43.5	7.7	-	27.5	58.0	88.7
Q4(24)@OE1	T478@HG1	T478@OG1	-	47.3	9.6	-	28.0	60.5	96.7
S477@OG	Q4(24)@HE22	Q4(24)@NE2	-	33.1	5.7	0.8	10.1	20.2	72.2
T478@OG1	Q4(24)@HE21	Q4(24)@NE2	-	3.2	6.7	-	-	-	-
T478@OG1	Q4(24)@HE22	Q4(24)@NE2	-	3.2	3.2	-	0.6	7.5	0.5
N487@OD1	R8(28)@HE	R8(28)@NE	-	-	-	-	-	5.4	2.6
N487@OD1	R8(28)@HH12	R8(28)@NH1	-	-	-	-	-	2.3	58.1
N487@OD1	R8(28)@HH21	R8(28)@NH2	-	-	-	-	-	24.2	3.9
N487@OD1	R8(28)@HH22	R8(28)@NH2	-	-	-	-	-	8.5	15.2

Acceptor[a]	DonorH[a]	Donor[a]	Hydrogen bond occupancy (%)						
			ACE2[36]	SBP1[36]	SPB25[36]	SPB25$_{K11F}$	SPB25$_{Q22R}$	SPB25$_{F8R/K11W/L25R}$	SPB25$_{F8R/K11F/Q22R/L25R}$
R8(28)@NH1	N487@HD22	N487@ND2	-	-	-	-	-	-	8.0
Y489@OH	R8(28)@HH11	R8(28)@NH1	-	-	-	-	-	8.9	-
D10(30)@OD1	K417@HZ1	K417@NZ	18.3	22.2	19.3	19.8	15.0	22.6	13.5
D10(30)@OD1	K417@HZ2	K417@NZ	18.0	27.2	28.9	10.7	20.1	17.4	20.8
D10(30)@OD1	K417@HZ3	K417@NZ	14.8	14.3	23.9	9.7	16.6	24.5	29.5
D10(30)@OD2	K417@HZ1	K417@NZ	24.2	24.8	16.6	23.3	14.8	20.1	14.0
D10(30)@OD2	K417@HZ2	K417@NZ	23.4	34.2	25.0	19.7	12.0	16.4	10.2
D10(30)@OD2	K417@HZ3	K417@NZ	16.4	19.7	21.4	17.0	17.1	26.0	11.9
Q493@OE1	K11(31)@HZ1	K11(31)@NZ	20.1	12.8	30.5	-	1.8	-	-
Q493@OE1	K11(31)@HZ2	K11(31)@NZ	20.8	14.3	32.9	-	1.8	-	-
Q493@OE1	K11(31)@HZ3	K11(31)@NZ	19.2	14.8	29.4	-	2.9	-	-
H14(34)@O	Q493@HE21	Q493@NE2	9.1	6.8	5.7	0.1	0.8	0.1	-
H14(34)@O	Q493@HE22	Q493@NE2	0.2	17.9	0.1	2.3	53.5	67.2	77.2
Q493@NE2	H14(34)@HD1	H14(34)@ND1	-	-	-	20.2	-	-	-
E15(35)@OE1	Q493@HE22	Q493@NE2	38.6	28.6	54.4	-	4.2	-	0.2
E15(35)@OE2	Q493@HE22	Q493@NE2	42.9	28.2	48.6	-	4.8	-	0.2
E17(37)@O	Y505@HH	Y505@OH	-	-	-	-	-	-	8.9
E17(37)@OE1	Q493@HE22	Q493@NE2	-	-	-	7.4	-	-	-
E17(37)@OE1	R403@HH11	R403@NH1	0.1	-	-	47.8	41.9	-	-
E17(37)@OE1	R403@HH12	R403@NH1	-	64.4	-	-	-	60.7	41.5
E17(37)@OE1	R403@HH21	R403@NH2	-	-	-	-	-	42.4	29.4
E17(37)@OE1	R403@HH22	R403@NH2	-	44.4	-	21.7	21.8	-	-
E17(37)@OE1	Y505@HH	Y505@OH	24.9	0.5	-	42.9	56.2	75.2	50.5
E17(37)@OE2	R403@HH11	R403@NH1	-	-	-	52.9	48.7	-	-
E17(37)@OE2	R403@HH12	R403@NH1	-	27.4	-	-	-	38.6	58.1
E17(37)@OE2	R403@HH21	R403@NH2	-	-	-	-	-	26.5	40.7
E17(37)@OE2	R403@HH22	R403@NH2	-	27.7	-	24.4	25.6	-	-
E17(37)@OE2	Y505@HH	Y505@OH	12.1	1.0	-	51.7	52.8	82.6	53.8
D18(38)@OD1	Q493@HE21	Q493@NE2	-	6.8	-	48.9	1.9	3.6	4.9
D18(38)@OD1	R403@HH12	R403@NH1	-	-	31.2	-	-	-	-
D18(38)@OD1	R403@HH22	R403@NH2	-	-	36.1	-	-	-	-
D18(38)@OD1	Y505@HH	Y505@OH	-	-	54.6	-	-	-	-
D18(38)@OD2	Q493@HE21	Q493@NE2	-	6.5	-	42.8	1.6	2.3	5.2
D18(38)@OD2	R403@HH12	R403@NH1	-	-	49.6	-	-	-	-
D18(38)@OD2	R403@HH22	R403@NH2	-	-	73.4	-	-	-	-
D18(38)@OD2	Y505@HH	Y505@OH	-	-	19.7	-	-	-	-
F20(40)@O	N501@HD21	N501@ND2	-	-	-	-	-	-	6.6
Y21(41)@O	G502@H	G502@N	-	99.7	-	-	-	-	-
Y21(41)@O	N501@HD21	N501@ND2	-	-	-	94.8	97.8	0.1	44.3

Acceptor[a]	DonorH[a]	Donor[a]	Hydrogen bond occupancy (%)						
			ACE2[36]	SBP1[36]	SPB25[36]	SPB25$_{K11F}$	SPB25$_{Q22R}$	SPB25$_{F8R/K11W/L25R}$	SPB25$_{F8R/K11F/Q22R/L25R}$
Y21(41)@O	Q498@HE22	Q498@NE2	-	-	-	57.8	24.1	-	-
Y21(41)@OH	G502@H	G502@N	-	-	84.4	-	-	-	-
Y21(41)@OH	N501@HD22	N501@ND2	17.5	-	5.3	-	-	-	-
Y21(41)@OH	Q498@HE21	Q498@NE2	5.5	-	-	-	-	-	-
Y21(41)@OH	T500@HG1	T500@OG1	19.5	-	-	-	-	-	-
Y505@O	Y21(41)@HH	Y21(41)@OH	-	-	-	98.3	99.1	95.8	99.3
G446@O	Q22(42)@HE22	Q22(42)@NE2	3.0	-	2.2	15.8	-	-	-
G446@O	R22(42)@HH11	R22(42)@NH1	-	-	-	-	27.7	-	-
G496@O	Q22(42)@HE22	Q22(42)@NE2	-	-	39.8	23.1	-	-	-
N448@O	R22(42)@HH12	R22(42)@NH1	-	-	-	-	65.0	-	-
N448@O	R22(42)@HH22	R22(42)@NH2	-	-	-	-	27.1	-	-
N501@ND2	Q22(42)@HE22	Q22(42)@NE2	-	-	6.9	-	-	-	-
N501@OD1	Q22(42)@HE22	Q22(42)@NE2	-	-	24.2	-	-	-	-
Q/R22(42)@O	Q498@HE21	Q498@NE2	-	-	0.8	6.8	0.5	23.5	-
Q/R22(42)@O	Q498@HE22	Q498@NE2	-	-	39.8	1.1	0.1	0.1	-
Q22(42)@NE2	N501@HD21	N501@ND2	-	-	11.8	-	-	-	-
Q22(42)@OE1	N501@HD21	N501@ND2	-	-	69.1	-	-	-	-
Q22(42)@OE1	Q498@HE21	Q498@NE2	0.5	-	27.1	5.7	-	-	-
Q22(42)@OE1	Y449@HH	Y449@OH	2.6	-	6.1	-	-	-	-
Q498@OE1	Q22(42)@HE21	Q22(42)@NE2	-	-	16.7	-	-	0.7	-
S494@OG	R22(42)@HH22	R22(42)@NH2	-	-	-	-	35.4	-	-
Y449@OH	Q22(42)@HE21	Q22(42)@NE2	5.3	-	4.7	1.4	-	-	-
Y449@OH	Q22(42)@HE22	Q22(42)@NE2	0.2	-	13.6	0.4	-	-	-
Y495@O	R22(42)@HH22	R22(42)@NH2	-	-	-	-	5.5	-	-
S23(43)@O	N501@HD22	N501@ND2	-	-	-	16.7	-	-	-
S23(43)@O	Q498@HE21	Q498@NE2	-	-	-	15.8	6.8	-	-
S23(43)@O	Q498@HE22	Q498@NE2	-	-	-	17.1	8.5	-	-
S23(43)@O	T500@HG1	T500@OG1	-	-	0.1	6.5	-	-	-
S23(43)@O	Y505@HH	Y505@OH	-	56.2	-	-	-	-	-
S23(43)@OXT	Y505@HH	Y505@OH	-	41.8	-	-	-	-	-
N501@OD1	S24(44)@HG	S24(44)@OG	-	-	-	-	-	5.8	5.7
Q498@OE1	S24(44)@HG	S24(44)@OG	-	-	-	0.4	6.2	-	1.6
S24(44)@O	G502@H	G502@N	-	-	-	0.1	-	20.3	-
S24(44)@OG	G502@H	G502@N	-	-	-	-	-	94.8	-
S24(44)@OG	N501@HD21	N501@ND2	-	-	-	-	-	-	15.6
S24(44)@OG	N501@HD22	N501@ND2	-	-	-	-	-	-	17.6
S24(44)@OG	T500@HG1	T500@OG1	-	-	0.1	6.3	2.3	-	0.1
T500@OG1	S24(44)@HG	S24(44)@OG	-	-	-	6.7	5.8	-	29.7
G446@O	R25(45)@HE	R25(45)@NE	-	-	-	-	-	-	10.5

Acceptor[a]	DonorH[a]	Donor[a]	Hydrogen bond occupancy (%)						
			ACE2[36]	SBP1[36]	SPB25[36]	$SPB25_{K11F}$	$SPB25_{Q22R}$	$SPB25_{F8R/K11W/L25R}$	$SPB25_{F8R/K11F/Q22R/L25R}$
G446@O	R25(45)@HH21	R25(45)@NH2	-	-	-	-	-	-	8.3
L/R25(45)@O	T500@HG1	T500@OG1	-	-	-	-	8.9	-	-
L/R25(45)@OXT	T500@HG1	T500@OG1	-	-	0.1	0.1	22.4	-	0.2
T500@O	L/R25(45)@H	L/R25(45)@N	-	-	-	24.7	-	-	-
T500@O	R25(45)@HH11	R25(45)@NH1	-	-	-	-	-	14.4	-
T500@OG1	L/R25(45)@H	L/R25(45)@N	-	-	-	2.0	20.2	-	-
N487@OD1	Y(83)@HH	Y(83)@OH	95.4	-	-	-	-	-	-
T500@O	N(330)@HD21	N(330)@ND2	10.4	-	-	-	-	-	-
G496@O	K(353)@HZ1	K(353)@NZ	13.8	-	-	-	-	-	-
G496@O	K(353)@HZ2	K(353)@NZ	18.5	-	-	-	-	-	-
G496@O	K(353)@HZ3	K(353)@NZ	24.4	-	-	-	-	-	-
K(353)@O	G502@H	G502@N	99.7	-	-	-	-	-	-
Q498@OE1	K(353)@HZ1	K(353)@NZ	19.5	-	-	-	-	-	-
Q498@OE1	K(353)@HZ2	K(353)@NZ	23.7	-	-	-	-	-	-
Q498@OE1	K(353)@HZ3	K(353)@NZ	15.8	-	-	-	-	-	-
D(355)@OD2	T500@HG1	T500@OG1	61.9	-	-	-	-	-	-

[a] The residue number of ACE2 is in parenthesis.
